# Supplementary material for: Confidence Is the Bridge between Multi-stage Decisions
Source: Curr Biol. 2016 Dec 5;26(23):3157–68. doi: 10.1016/j.cub.2016.10.021 (PMC5154755; doi:10.1016/j.cub.2016.10.021)
Supplement: Document S1. Figures S1–S5, Tables S1 and S2, and Supplemental Experimental Procedures [file mmc1.pdf]

Current Biology, Volume *26*

Supplemental Information

Confidence Is the Bridge between Multi-stage Decisions

Ronald van den Berg, Ariel Zylberberg, Roozbeh Kiani, Michael N. Shadlen, and Daniel M. Wolpert

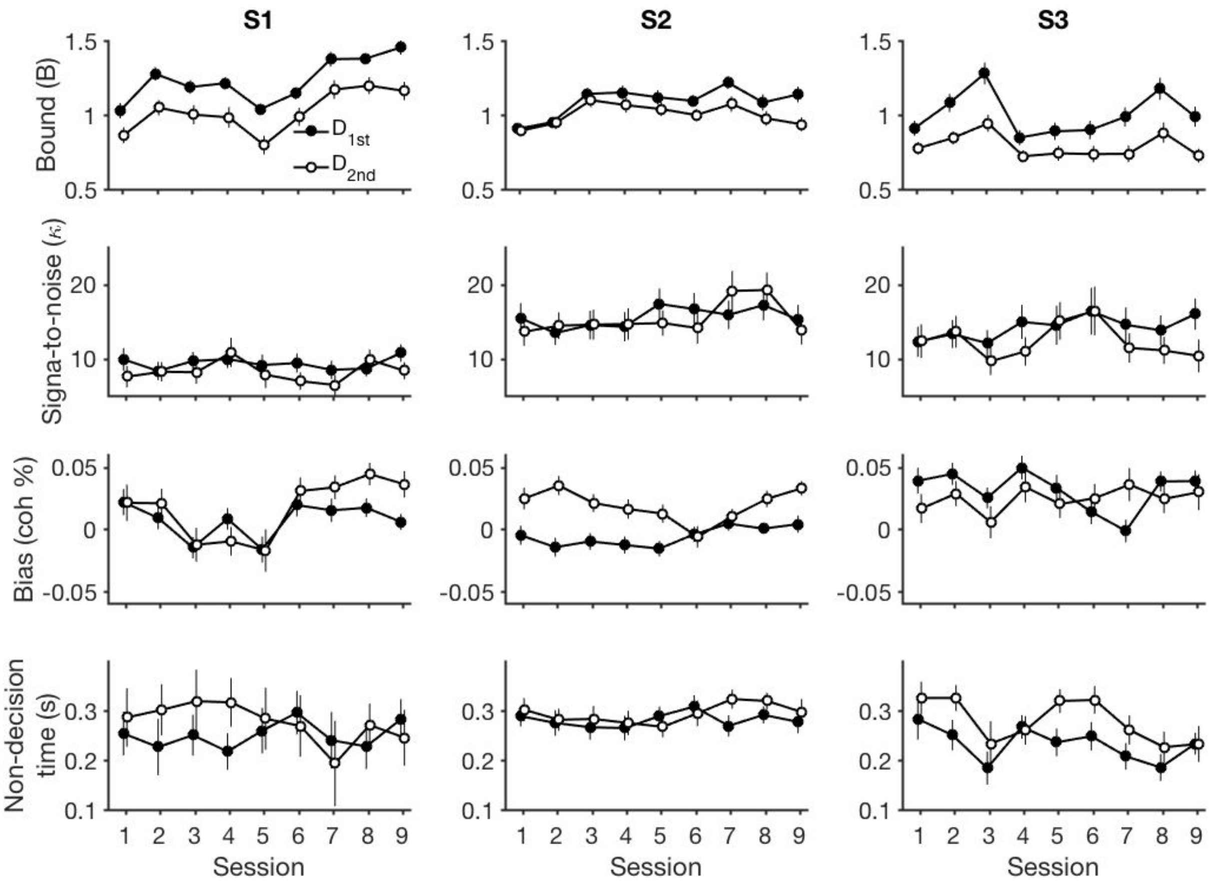

Figure S1. Drift diffusion parameter variation over sessions; Related to Figure 2. The drift-diffusion model was fitted separately to the D_{1st} and D_{2nd} decisions of double-decision trials for each session. Columns are participants and error bars show 95% confidence intervals for parameters estimates.

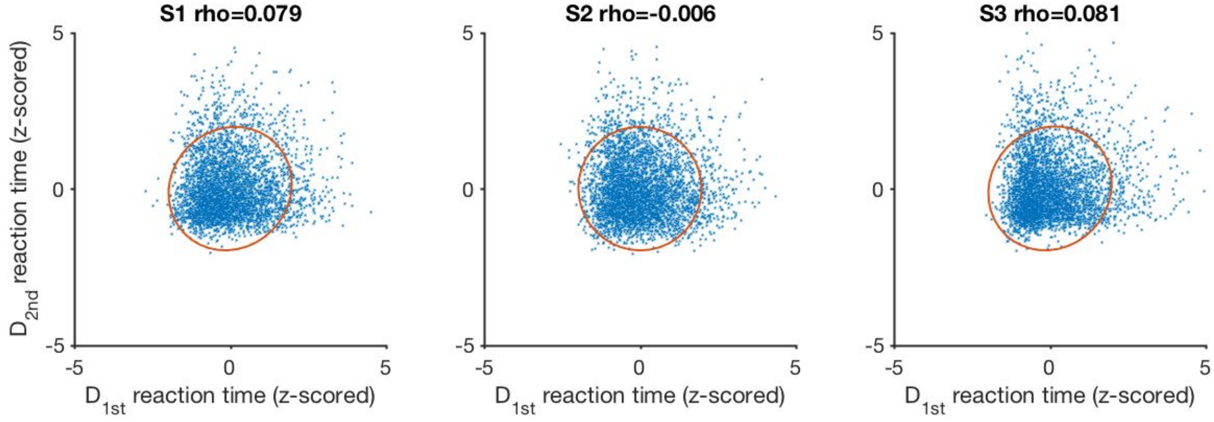

Figure S2. Correlation between reactions times on D_{1st} and D_{2nd}; Related to Figure 3. Reaction times were z-scored within each coherence and session. Also shown are 2-sd principal component ellipses. The correlations are significant for S1 ($p<0.001$) and S3 ($p<0.001$) but not for S2 ($p=0.68$).

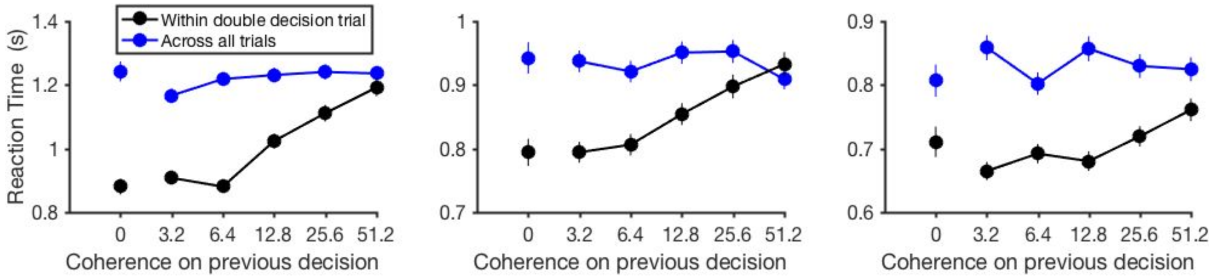

Figure S3 Within vs. across trial effects of coherence on previous decision vs. reaction time on current decision; Related to Figure 3. The black data reproduces Figure 3B (top), showing RT on D_{2nd} and a function of coherence on D_{1st}. The blue data show across-trial effects, showing reaction time on first decision of a trial against the coherence of the last decision on the previous trial. Coherence of the preceding trial decision does not affect the RT of the current decision ($p>0.19$ for all three subjects). Error bars show s.e.m.

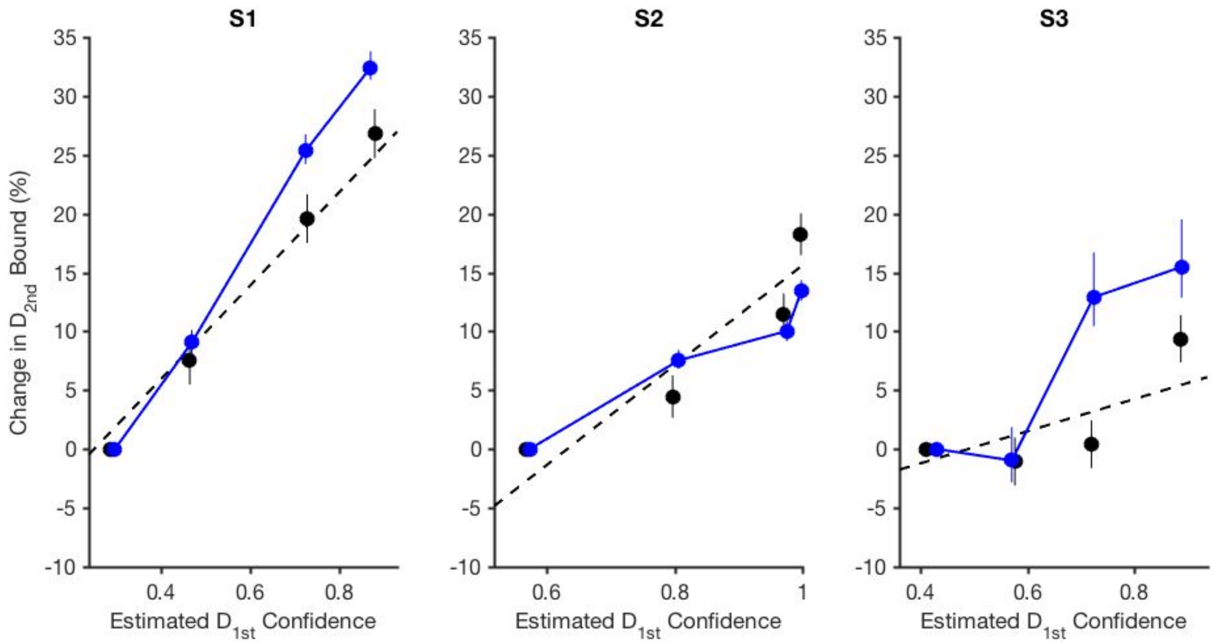

Figure S4. Estimates of the change in the bound on the second decision as a function of the estimated confidence about the first decision; related to Figure 5. The black data and lines are as for Figure 5 but with the model 3 refit for four quantiles. The blue points are derived from the D_{2nd} and D_{2*} 0% coherence trials. On these trials RT is determined by bound height and non decision time ($E[RT]=B^2 + t_{nd}$). For each session we calculated mean RT for each quartile of estimated D_{1st} confidence (quartiles splits across all trials) for these trials and derived the bound height (using t_{nd} =0.280 s, approximately the mean across subjects; Table S2). We plot the across-session mean of the estimated change in second decision bound. Error bars (s.e.) were derived by bootstrapping (1,000 samples).

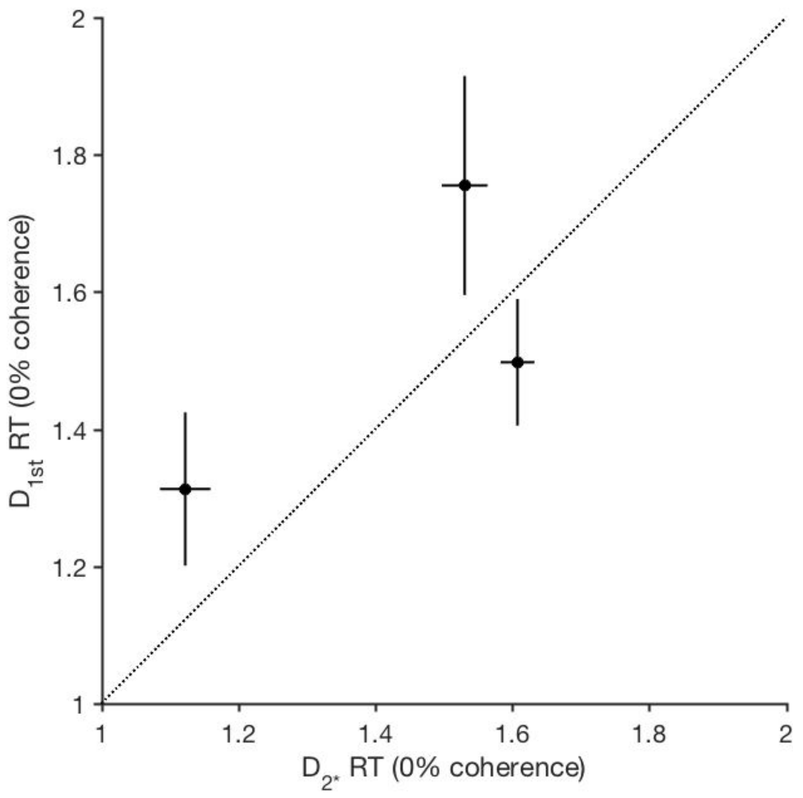

Figure S5. RT on first decisions against second decision; related to Figure 3. Data shows mean (±s.e.) for 0% coherence trials for each subject. The dotted line shows RT equality.

Subject	Trials	Bound (B)	Signal-To-Noise (κ)	Non-decision time (t_{nd}, s)
S1	D_{1*}	1.25 ± 0.03	9.2 ± 0.7	0.242 ± 0.026
	D_{1st}	1.25 ± 0.01	9.1 ± 0.2	0.243 ± 0.008
S2	D_{1*}	1.14 ± 0.02	11.9 ± 0.9	0.250 ± 0.018
	D_{1st}	1.07 ± 0.01	15.1 ± 0.3	0.281 ± 0.004
S3	D_{1*}	1.02 ± 0.04	11.7 ± 0.9	0.193 ± 0.016
	D_{1st}	0.95 ± 0.01	12.4 ± 0.3	0.222 ± 0.005

Table S1. Fitted parameters of the drift-diffusion model; related to Figure 2. The model was fit to single first decisions (D_{1*}) and to the first of two decisions (D_{1st} including $D_{1st\text{-}catch}$ trials). Parameter means are shown ±s.e. For simplicity bias (C_0) was set to zero.

			DOF	ΔBIC from best model		
Model	Bound (B)	Signal-to-noise (κ)		S1	S2	S3
1	$B_{session}$	κ	12	190.83	125.2	5.4
2	B	$\kappa_{session}$	12	409.7	164.5	11.9
3	$B_{session} + \alpha \cdot conf$	κ	13	0.0	0.0	0.0
4	B	$\kappa_{session} + \alpha \cdot conf$	13	364.7	135.9	14.2
5	$B_{session}$	$\kappa + \alpha \cdot conf$	13	140.9	92.1	8.3
6	$B + \alpha \cdot conf$	$\kappa_{session}$	13	227	46.9	4.8
	MLE parameters for Model 3					
		α	1	0.355 ± 0.026	0.379 ± 0.034	0.103 ± 0.028
		$\mathbb{E}[B_{session}]$	9	0.791 ± 0.026	0.678 ± 0.034	0.713 ± 0.025
		κ	1	8.04 ± 0.23	15.44 ± 0.33	11.38 ± 0.33
		C_0	1	0.020 ± 0.002	0.018 ± 0.001	0.026 ± 0.002
		t_{nd} (s)	1	0.283 ± 0.009	0.297 ± 0.003	0.269 ± 0.005

Table S2. Model comparison for fits to the second decision of a double-decision trial (D_{2nd}); related to Figures 5 & 6. The models vary in whether and how they allow the bound (B) and κ for the second decision to vary (see Methods for details). Some models have different levels of B or κ for each of the 9 sessions (subscripts) and others allow these parameter to vary linearly with the predicted confidence from D_{1st} (conf). The degrees of freedom (DOF) of the models and their difference in BIC from the best model are shown. The maximum likelihood estimates of the parameters for the best model (3) are also shown with s.e.

Supplemental Experimental Procedures

Starting point vs. drift bias

We accounted for possible biases by including a bias on drift in the model (the C_0 parameter). However, there is some evidence suggesting that the locus of bias is instead in bound asymmetries (Refs S1, S2), (but see S3) which is equivalent to a starting point bias in our model. We compared these alternatives by fitting all first choices that were part of a double decision (D_{1st}), with either a C_0 (coherence bias) term or y_0 (offset bias) term. For all three subjects, the model with C_0 bias was strongly preferred over the model with y_0 (ΔBIC is 21.8, 21.1, and 183.0 for subjects 1-3, respectively; same as deviance as d.f. are same), which justifies the assumption in our main model. Note that S3 is the most informative subject as bias is small for S1 and S2. We chose to fit with only C_0 to reduce the number of parameters.

Normative model

We used dynamic programming to determine the optimal decision policy for D_{2nd} as a function of the confidence in D_{1st}. By optimal we refer to the decision policy that maximizes reward rate (i.e., maximizing the number of points obtained per unit of time). The goal of this exercise is not to establish that our participants were maximizing reward rate, but to justify their strategy as sensible given a cost of time per trial.

The random dot motion discrimination task can be considered an instance of a class of problems referred to as partially-observable Markov Decision Process (POMDP). The partial observability derives from the fact that (motion) observations provide only ambiguous evidence about the true underlying task state. Following the usual approach, we solve the POMDP casting it as a fully observable Markov decision process (MDP) over the belief states of the agent. We then use dynamic programming to find the policy that maximizes average reward.

Formally, an MDP can be described as a tuple given by (S4):

(i) a non-empty state space S,

(ii) an initial state S_0,

(iii) a goal state S_G,

(iv) a set of actions $A(s)$ applicable in state s,

(v) positive and negative rewards $r(a,s)$ for doing action a in state s,

(vi) transition probabilities $P_a(s'|s)$ indexing the probability of transitioning to state s' after doing action a in state s.

For simplicity, we derive the optimal policy for the second decision assuming that D_{2nd} is informed by the confidence in D_{1st}, without explicitly modeling the decision process for the first decision. Next, we describe how to cast the motion discrimination task as an MDP.

The state s was defined as a tuple $\langle x, t, c_1 \rangle$, where x is the amount of accumulated motion evidence for one direction and against the other (the decision variable). It is positive when the evidence supports one motion direction (say upwards), and negative when it supports the opposite direction. t is the elapsed decision time since the onset of motion for the second decision. c_1 is the probability that the first decision was correct. We assume that c_1 takes a value from the set $C_1 = [0.6, 0.8, 1]$, which corresponds respectively to the average confidence for incorrect, correct and bypassed first decisions. This is a simplification because correct and incorrect decisions are associated with a distribution of confidence values. However, we note that our conclusions do not depend on this simplification as long as the average confidence about D_{1st} is higher for correct than for error trials, which is indeed what was observed in our data (**Figure 2** and **4**). Further, we assume that the probability of eliciting each of the values in C_1 was given by $p_{C_1} = [0.3, 0.5, 0.2]$. Again, our conclusions are robust to changes in these values.

The decision process starts with $x = 0$ (i.e., no accumulated evidence favoring either of the alternatives), $t = 0$ and $c_1 \in C_1$. The distribution over c_1 was implemented with an initial state s_0 that has transition probabilities p_{c_1} to the three states $\langle x = 0, t = 0, c_1 \in C_1 \rangle$.

Three actions were applicable in each state. The decision maker could either terminate the trial by selecting one of the targets (two possible actions), or maintain fixation (the third 'action') to gather additional motion evidence. Defining a deterministic policy entails specifying which action to select in each state.

Transition probabilities $P_a(s'|s)$ indicate the probability of transitioning to s' after performing action a in state s. State transitions are not deterministic because they depend on the momentary motion evidence, which is stochastic even if the motion coherence were known. As in the bounded accumulation model, we assume that the momentary motion evidence follows a normal distribution with a mean that depends linearly on motion coherence, such that over

one second of stimulus viewing the evidence accumulated is, on average, $\kappa.coh$ and the variance of the momentary is equal to 1. For the analyses shown in **Figure 7** we set κ=10.

For a given motion coherence, the probability of transitioning from state $s = \langle x, t, c_1 \rangle$ to state $s' = \langle x', t+\delta t, c_1 \rangle$ is given by:

$$p_{fix}(s'|s, coh) = p_{fix}(\langle x', t + \delta t, c_1 \rangle \mid \langle x, t, c_1 \rangle, coh) = \mathcal{N}(x' - x | \kappa.coh.dt, \sqrt{\delta t}) \quad (1)$$

where $N(\cdot|\mu, \sigma)$ is the normal p.d.f. with mean μ and standard deviation σ.

Because the decision-maker does not know the motion coherence with certainty, obtaining the transition probability $p_{fix}(s' \mid s)$ requires marginalizing over coherences:

$$p_{fix}(s'|s) = \sum_{coh} p_{fix}(s'|s, coh) p(coh|s) \quad (2)$$

This marginalization requires knowledge of $p(coh|s)$, the probability that the underlying motion coherence is coh given that state s was reached (S5):

$$p(coh| \langle x, t, c_1 \rangle) = \frac{1}{\mathcal{Z}} \mathcal{N}(x | \kappa.coh.t, \sqrt{t}) p(coh) \quad (3)$$

where the coherences coh are the discrete set of signed coherences used in the experiment, and Z is the normalization constant which assures that the sum of $p(coh|\langle x, t, c_1 \rangle)$ over all motion coherences adds to one. As in the experiment, $p(coh)$ is distributed uniformly over the discrete set of motion coherences.

We assume that the optimal decision-maker maximizes the reward per unit of time. To find the optimal policy, we used value iteration to solve Bellman's equation. For problems that have a recurrent state—which includes decision-making tasks that are organized as a sequence of trials—the problem of maximizing average reward can be recast as a stochastic shortest path problem (or Goal MDP) through the inclusion of an artificial cost-free and absorbing goal state (S4). The intuition behind this simplification is that if we consider a sequence of generated

trajectories in state space, we can divide it into a series of visits to the recurrent state, which is equivalent to the corresponding Goal MDP where the recurrent state is the goal state (S4).

For our task, the Bellman equation takes the form:

$$V(s) = max \begin{cases} Q(s, up) & = p_{c|up}(s)R_c + p_{nc|up}(s)R_{nc} - (t_{other} + t_{nd})\rho \\ Q(s, down) & = p_{c|down}(s)R_c + p_{nc|down}(s)R_{nc} - (t_{other} + t_{nd})\rho \\ Q(s, fix) & = E[V(s')|s] - \rho\delta t \end{cases} \quad (4)$$

where s , s' are states and t_{nd} is the average non-decision time. The time t_{other} is the average time gap between a response to D_{2nd} and the onset of motion for the following D_{2nd}; it includes the time spent on fixations, reporting confidence, responding to the first decision, receiving feedback, etc. For the analyses of **Figure 7**, we chose $t_{nd} = 0.3\ s$ and $t_{other} = 5\ s$. R_c is the reward obtained after a correct response, and R_{nc} is the reward obtained after an incorrect response. As in the experiment, $R_c = 1$ and $R_{nc} = 0$. Reward rate ρ is the reward obtained per unit of time.

$p_{c|a}(s)$ is the probability of being correct after doing action a in state s . For the double decision trials, being correct means solving both decisions correctly. Therefore, $p_{c|a}(s)$ is the probability that the first decision was correct (c_1) , multiplied by the probability that D_{2nd} was solved correctly. The latter can be obtained summing over the coherences for which the action a is the appropriate action. For instance, the action 'up' is the appropriate action for all positive and for half of the 0% coherence trials. Therefore

$$p_{c|up}(s) = p_{c|up}(x, t, c_1) = c_1 \times \left(\sum_{coh>0} p(coh|x, t) + \frac{1}{2} p(coh = 0|x, t) \right) \quad (5)$$

Our depiction of Bellman's equation implicitly assumes that choosing a terminal action leads to an absorbing cost-free state. The expectation in $Q(s, fix)$ is an expectation over all future states s' that result from being in s and gathering evidence for an additional time step δt :

$$E[V(s')|s] = \int_{s' \in S} ds' p_{fix}(s'|s) V(s') \quad (6)$$

Because time flows in a single direction, Bellman's equation can be solved by backwards induction in a single pass. However, since we want to maximize the reward rate, which depends

on the policy itself, we perform multiple backwards passes to find the value of ρ through root-finding, bracketing ρ within a sequence of diminishing intervals until the value of the initial state $V(S_0)$ is approximately zero (S4, S6).

Supplemental References

S1. White, C. N., and Poldrack, R. A. (2014) Decomposing bias in different types of simple decisions. J. Exp. Psychol. Learn. Mem. Cogn. 40, 385–398

S2. van Ravenzwaaij, D., Mulder, M. J., Tuerlinckx, F., and Wagenmakers, E.-J. (2012) Do the dynamics of prior information depend on task context? An analysis of optimal performance and an empirical test. Front. Psychol. 3, 132

S3. Moran, R. (2015) Optimal decision making in heterogeneous and biased environments. Psychon. Bull. Rev. 22, 38–53

S4. Bertsekas, D. P. (1995) Dynamic programming and optimal control (Athena Scientific, Belmont, MA)

S5. Moreno-Bote, R. (2010) Decision confidence and uncertainty in diffusion models with partially correlated neuronal integrators. Neural Comput. 22, 1786–1811

S6. Drugowitsch, J., Moreno-Bote, R., Churchland, A. K., Shadlen, M. N., and Pouget, A. (2012) The cost of accumulating evidence in perceptual decision making. J. Neurosci. 32, 3612–3628
