# Supplementary material for: Confidence Is the Bridge between Multi-stage Decisions
Source: Curr Biol. 2016 Dec 5;26(23):3157–68. doi: 10.1016/j.cub.2016.10.021 (PMC5154755; doi:10.1016/j.cub.2016.10.021)
Supplement: Document S2. Article plus Supplemental Information [file mmc2.pdf]

Article

Current Biology

Confidence Is the Bridge between Multi-stage Decisions

Graphical Abstract

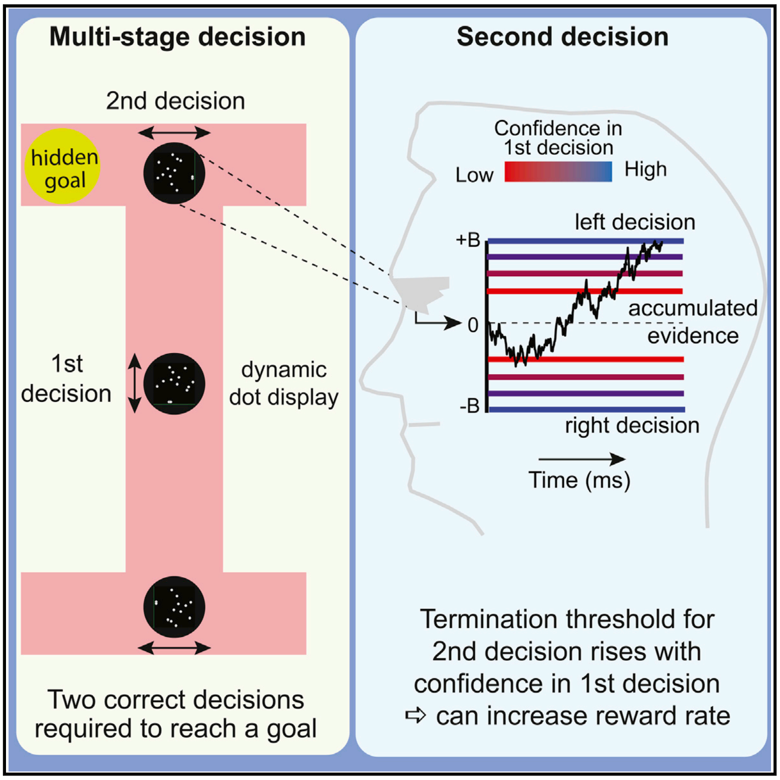

Authors

Ronald van den Berg, Ariel Zylberberg, Roozbeh Kiani, Michael N. Shadlen, Daniel M. Wolpert

Correspondence

wolpert@eng.cam.ac.uk

In Brief

van den Berg et al. show that when making a sequence of decisions to achieve a goal, the subjective confidence in the accuracy of the first decision precisely and rapidly alters the decision-making process of the second decision.

Highlights

- Many tasks require a series of correct decisions to reach a goal
- Confidence in a decision affects the termination criterion for the next decision
- Use of confidence to change the speed-accuracy trade-off can increase reward

van den Berg et al., 2016, Current Biology *26*, 3157–3168
December 5, 2016 © 2016 The Author(s). Published by Elsevier Ltd.
http://dx.doi.org/10.1016/j.cub.2016.10.021

CellPress

Current Biology
Article

Confidence Is the Bridge between Multi-stage Decisions

Ronald van den Berg,[1,4] Ariel Zylberberg,[2,4] Roozbeh Kiani,[3] Michael N. Shadlen,[2] and Daniel M. Wolpert[1,5,*]
[1]Computational and Biological Learning Laboratory, Department of Engineering, Cambridge University, Cambridge CB2 1PZ, UK
[2]Department of Neuroscience, Zuckerman Mind Brain Behavior Institute, Kavli Institute of Brain Science, and Howard Hughes Medical Institute, Columbia University, New York, NY 10032, USA
[3]Center for Neural Science, New York University, New York, NY 10003, USA
[4]Co-first author
[5]Lead Contact
*Correspondence: wolpert@eng.cam.ac.uk
http://dx.doi.org/10.1016/j.cub.2016.10.021

SUMMARY

Demanding tasks often require a series of decisions to reach a goal. Recent progress in perceptual decision-making has served to unite decision accuracy, speed, and confidence in a common framework of bounded evidence accumulation, furnishing a platform for the study of such multi-stage decisions. In many instances, the strategy applied to each decision, such as the speed-accuracy trade-off, ought to depend on the accuracy of the previous decisions. However, as the accuracy of each decision is often unknown to the decision maker, we hypothesized that subjects may carry forward a level of confidence in previous decisions to affect subsequent decisions. Subjects made two perceptual decisions sequentially and were rewarded only if they made both correctly. The speed and accuracy of individual decisions were explained by noisy evidence accumulation to a terminating bound. We found that subjects adjusted their speed-accuracy setting by elevating the termination bound on the second decision in proportion to their confidence in the first. The findings reveal a novel role for confidence and a degree of flexibility, hitherto unknown, in the brain's ability to rapidly and precisely modify the mechanisms that control the termination of a decision.

INTRODUCTION

Difficult decisions arise through a process of deliberation involving the accumulation of evidence acquired over time. They thus invite a trade-off between speed and accuracy, instantiated as a rule for terminating the decision and committing to a choice [1, 2]. The speed-accuracy trade-off established through this rule is influenced by the cost of time weighed against the reward for an accurate decision and the penalty for an error [3–5]. In many instances, the regime is established through instruction, expertise, or some broad optimization goal, such as maximizing reward over time. In less certain environments, however, decision policy may benefit from adjustment on a shorter timescale [6–8]. For example, when a decision maker must complete two (or more) choices to achieve a goal, the policy applied on the second choice might be adjusted based on the prediction about the success of the first decision. These types of multi-stage decisions arise in foraging, exploration, and structured reasoning (e.g., [9, 10]).

Recent studies of single-stage perceptual decisions have served to unite decision accuracy, speed, and confidence in a common framework of bounded evidence accumulation [11–14]. The quantitative features of this model system provide a framework for studying multi-stage decisions. In a well-studied motion discrimination task, the decision itself (e.g., up or down) is governed by the accumulation of noisy samples of evidence from the visual stimulus and transduced by sensory neurons [15, 16]. The accumulation is represented by neurons in the association cortex such that their firing rate is proportional to the accumulated evidence for one choice versus the other. This representation, termed a decision variable, is compared to a threshold (i.e., bound), which terminates the decision process, thereby establishing both the choice and decision time. The latter corresponds to the measured reaction time, but there are processing delays that separate these events by enough time to allow for a dissociation between the state of accumulated evidence used to terminate the decision and the evidence used to support subsequent behaviors, including a change of mind [17–19].

Confidence is informed by an implicit mapping between the state of the neural representation of accumulated evidence used to make the decision and the likelihood that it would support a correct choice [11, 20]. Since confidence can also undergo revision after commitment [13, 21, 22], it is possible for a subject to make a decision and believe that she made an error. Confidence thus conforms to an internal prediction about the success or failure of one's decisions. Often when a sequence of multiple decisions are required to achieve a single goal, the success of each decision is not known until the goal is reached, if ever. Therefore, as accuracy is not known, confidence is likely to play an important role in situations that require a sequence of decisions to reach a goal.

Here we test the hypothesis that confidence is carried forward from a decision to control the speed-accuracy trade-off of a second decision. Subjects made a multi-stage decision that

© 2016 The Author(s). Published by Elsevier Ltd.
This is an open access article under the CC BY license (http://creativecommons.org/licenses/by/4.0/).

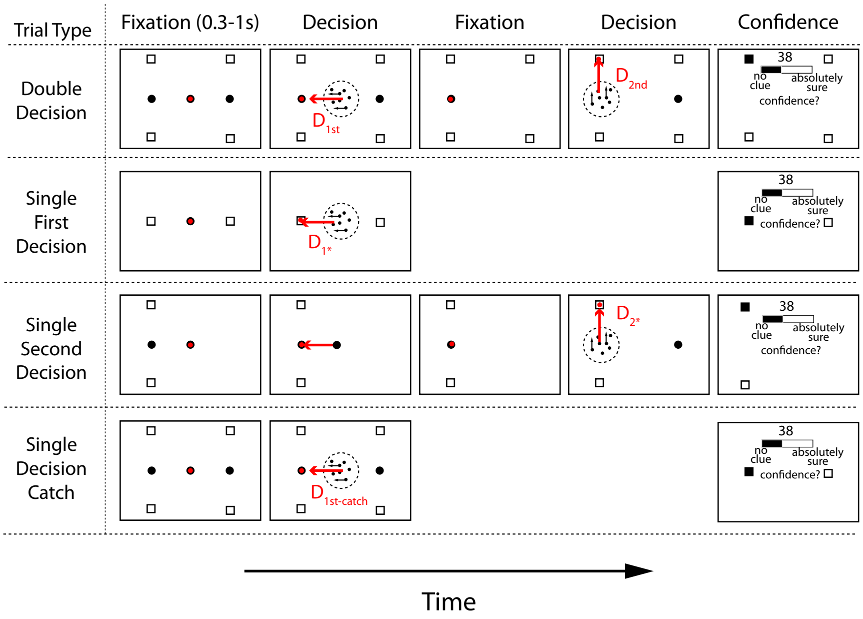

Figure 1. Experimental Paradigm
All trials started with central fixation (red circles show eye position). On *double-decision* trials, subjects judged the perceived direction of motion (left versus right) of a central random-dot display and, whenever ready, made an eye movement to one of two corresponding choice targets. After fixation, a second random-dot display appeared, and subjects judged the perceived direction of motion (up versus down) and made an eye movement to the corresponding final-choice target. After indicating their confidence that both decisions were correct, they received feedback on whether the selected final-choice target was correct. On *single first decision* trials, only the left-right choice targets were displayed and a single first decision was required, followed by the confidence judgement. On *single second decision* trials, only one lateral choice target and the corresponding final-choice targets were displayed. Subjects made an eye movement to the lateral choice target, mimicking a first decision. Then the motion display appeared, leading to D_2*. On *single-decision catch* trials, the setup was the same as on double-decision trials, but all targets disappeared after the first decision and subjects then made a confidence judgement and the trial was terminated. Note that for visualization purposes, stimuli in this figure are not to scale and the confidence bar is not displayed at its true location (see Experimental Procedures).

involved two perceptual decisions separated briefly in time, and success required both decisions to be correct. We measured choice and reaction time of both, and we extracted an estimate of confidence in the first decision. Subjects elevated their termination criterion on the second decision in proportion to their confidence in the first decision. Therefore, when they were more confident in their first decision, they took more time and were more accurate on their second decision, choosing a more conservative termination criterion when building on a successful foundation. We show that this strategy is rational if the time to make a decision is costly. Our results therefore point to a more general capacity to adjust decision-making on a fast timescale, based on the confidence one has in a previous decision.

RESULTS

Three naive subjects were asked to decide about the net direction of motion in a dynamic random-dot display (Figure 1). Both the direction (e.g., left or right) and the strength of motion were random from trial to trial, and the subjects indicated their decision by making an eye movement to one or the other *choice target*, whenever ready, thereby providing a measure of reaction time. The random dot display was extinguished once the eye movement was initiated. On most trials (Figure 1 top row), the first decision (D_{1st}) led to the display of a new random dot display, centered at the location of the first chosen target. The subject was then required to make a second decision (D_{2nd}) about the direction of motion (up or down), again indicated by an eye movement, when ready. The direction and motion strength of D_{1st} and D_{2nd} were both random and independently chosen. Feedback was provided only after both decisions were made. If either choice was an error, the entire sequence was designated as such. In other words, both decisions were required to be correct for success on the trial (see Experimental Procedures). These double-decision trials (D_{1st} then D_{2nd}) constituted 79% of the trials. The others comprised a variety of single decisions (Figure 1), most of which were explicitly cued as such. Subjects thus knew that success on these trials rested on just one correct decision. Subjects performed a fixed number of trials each session.

All three subjects made faster and more accurate decisions when they viewed stronger motion. Figure 2 illustrates these trends in the data for the first decision. The data are well captured by a bounded drift-diffusion model (smooth curves), as previously shown [15, 23, 24]. On trials in which we did not present a second stimulus, we obtained a confidence rating after the subject indicated their choice (Figure 2, bottom row) but before receiving feedback. Not surprisingly, decisions were associated with greater confidence if they were correct, with the highest confidence associated with the strongest motion. Note that the confidence rating was obtained after the choice (cf. [12]) and therefore likely benefited from information in the display that did not arrive in time to affect the choice [13]. We hypothesized that confidence about the first decision could bear on the strategy used to make a second decision. We next consider the changes in strategy that arise when making two decisions in sequence, beginning with the first decision.

First of Two Decisions

Faced with a decision, it is possible that a decision maker might apply a different strategy if she knows ahead of time that this is the first of a sequence or the sole decision that will affect outcome. To evaluate this possibility, we examine choice accuracy and reaction times (RTs) under conditions in which the subject was explicitly instructed that they would make only a single first decision (Figure 2, blue curves) and compared these

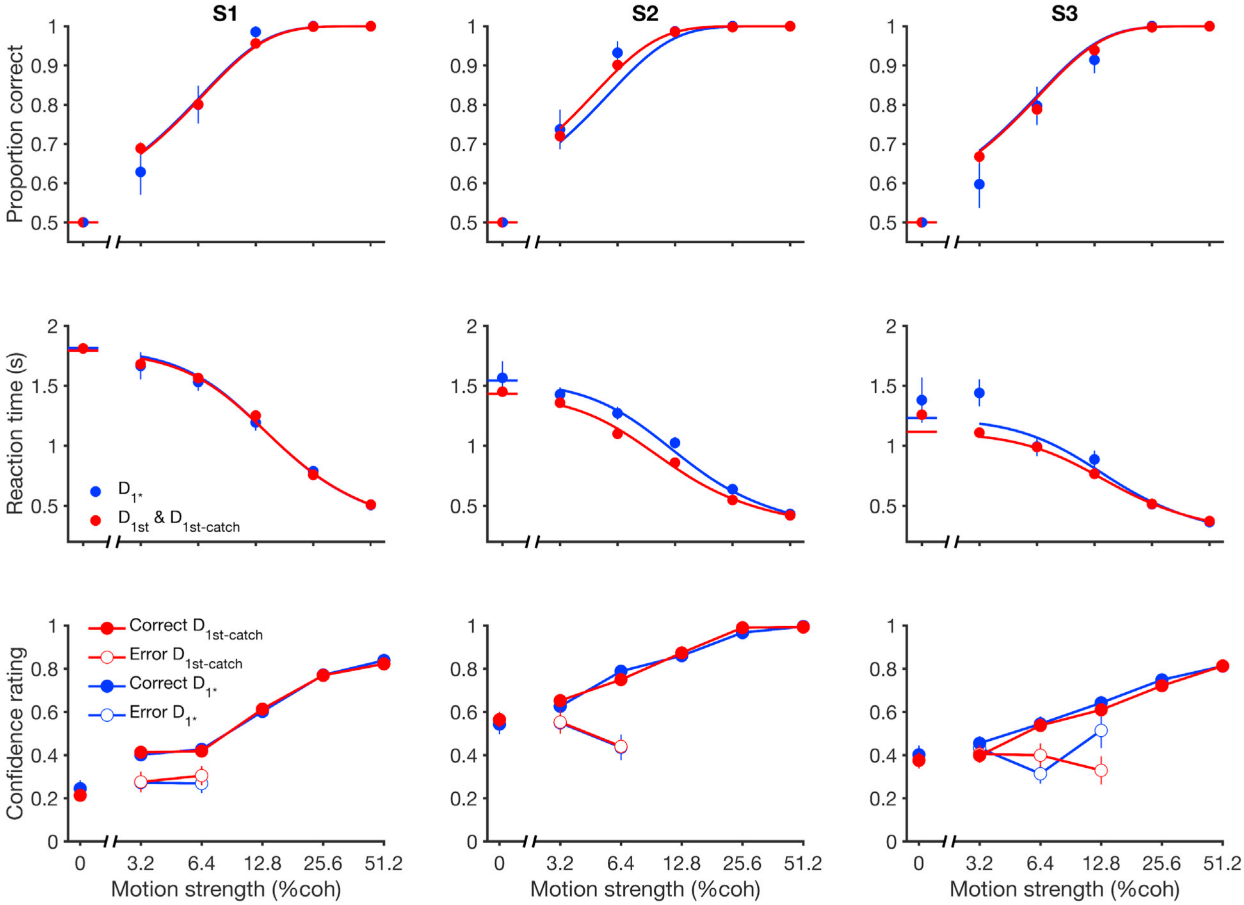

Figure 2. Accuracy, Reaction Time, and Confidence for First Decisions

The top and middle rows show the proportion of correct decisions and reaction times as a function of motion strength on single first decisions (blue: D_1* trials) and on first decisions on trials in which subjects made (or thought they would make) two decisions (red: D_{1st} and $D_{1st\text{-}catch}$ trials). Solid lines are fits of a drift-diffusion model to each dataset. The bottom row shows the confidence ratings on correct (filled) and error (open) trials for both single first decision (blue: D_1*) and single-decision catch trials (red: $D_{1st\text{-}catch}$). Note that 0% trials have been designated as correct for plotting. Columns S1–S3 correspond to individual subjects. Error bars show SEM. See also Figure S1 and Table S1.

to performance when the subject believed that the decision was the first of two (Figure 2, red curves). We observed only subtle differences in decision accuracy, which were not statistically reliable ($p > 0.36$). Two subjects exhibited shorter RTs on the single-decision trials (reduction of S2: 90 ms; $p < 0.001$; reduction of S3: 60 ms; $p = 0.025$ ANOVA). The drift-diffusion model attributes this to a small change in κ and non-decision time (Table S1). Note that this difference is not explained by a change in the termination criteria, that is, the bound height, which would lead to larger differences in the RTs at the lower coherences—a pattern that will be apparent in the next section.

From this analysis, we are unable to draw strong conclusions about a change in decision policy induced by the need to make two decisions in sequence. The data do not rule out this potential strategy, but it was not exercised to great effect in this experiment. The observation is mainly interesting when contrasted with the subjects' adjustments to their decision criteria in the second of two decisions. It will also prove convenient when we exploit confidence ratings from the single first decisions later on.

Second of Two Decisions

Both the accuracy and RT of the second decision depended on the experience of the first decision (Figure 3). For example, if the first decision resulted in an error, subjects were faster and less accurate on their second decision (Figure 3A, red traces) than they were if the first decision was correct (blue traces). The breakdown of the second decision by whether the first was correct or an error implies that aspects of the first decision may affect the second decision. However, as subjects did not receive feedback until completion of the two decisions, they could not know if they were correct or not when they entered the second decision. We hypothesized that they carried forward their confidence after the first decision—an internal prediction or belief that they were correct [6, 11, 25]—to adjust criteria applied to make the second decision.

Before evaluating this hypothesis in detail, it is important to consider plausible alternatives. Specifically, slow fluctuations in attention or any other factors that affect the speed-accuracy trade-off on both the first and second decisions could produce an association between the accuracy of the first decision and

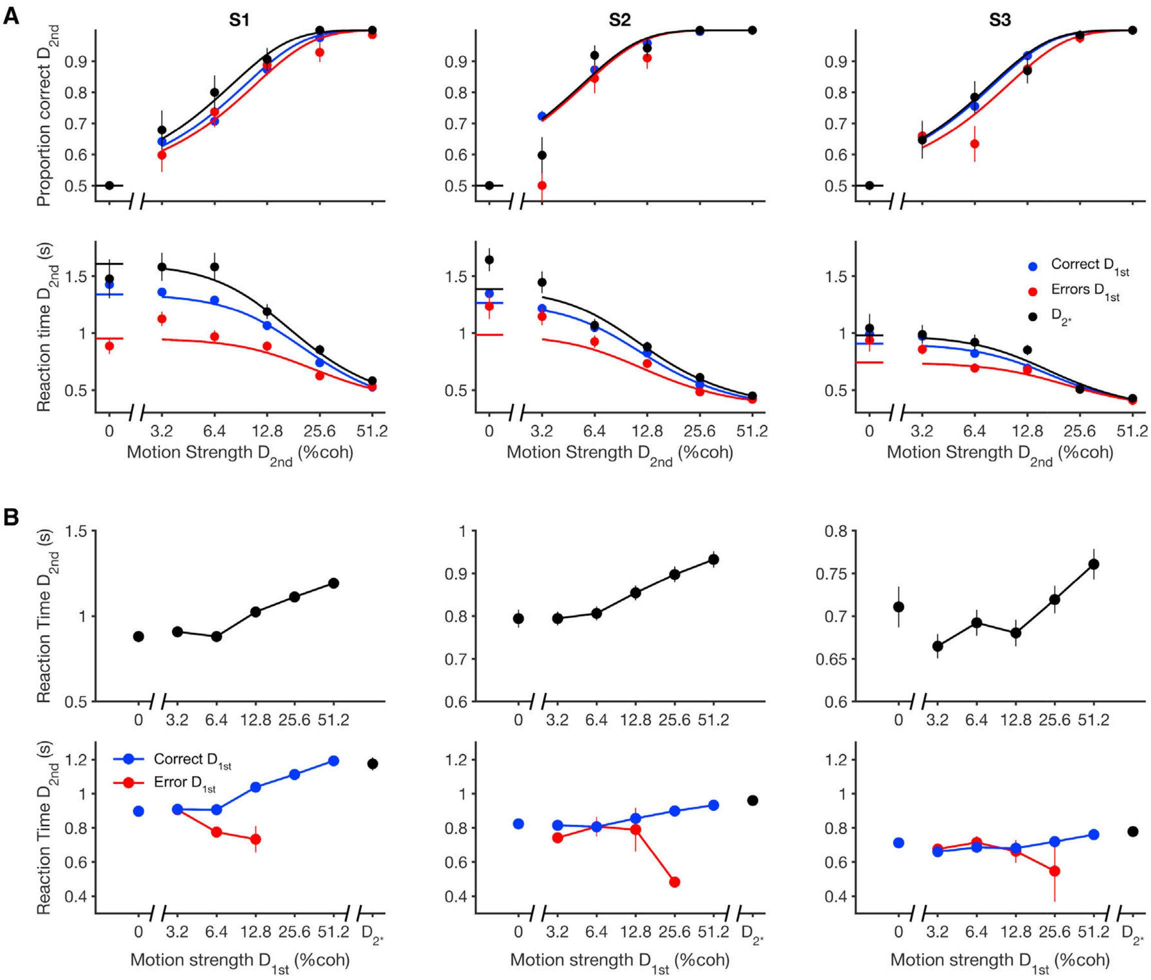

Figure 3. Accuracy and Reaction Time for Second Decisions

(A) Data plotted against motion strength on D_{2nd}. Trials are split by whether the first decision was correct (blue) or an error (red). The black data points are for single second decisions (D_2*). Columns S1–S3 correspond to individual subjects, and solid lines are fits of a drift-diffusion model to each dataset.

(B) Reaction time plotted as a function of the motion strength of the first decision (D_{1st}) for all trials (top) and split (bottom) by whether the D_{1st} decision was correct (blue) or an error (red). The final black data points are for trials with a single second decision (D_2*).

Error bars show SEM. See also Figures S2, S3, and S5.

performance on the second. We know that such fluctuations exist in our data (Figures S1 and S2). Therefore, by selecting error trials, we might have also selected trials in which the second decision tended to be faster and less accurate due to a common cause (fluctuations in the decision-making process across the two decisions). However, co-fluctuations cannot explain three additional observations. First, the difficulty (motion strength) of D_{1st} affected both the accuracy ($p < 0.01$ for all subjects) and reaction times on D_{2nd} (Figure 3B top; $p < 0.0001$ for all subjects). The difficulty is independent of any such co-fluctuations because the motion strength of D_{1st} and D_{2nd} were uncorrelated.

Second, subjects performed single decisions similarly to the second of two decisions preceded by the easiest motion strength. We examined a set of trials in which subjects made just one decision using the identical task geometry as the second of a sequence of decisions (labeled D_2* in Figure 1). As these decisions are not selected based on the performance on a previous decision, the effect of fluctuations should produce RTs represented by a mixture of the D_{2nd} RTs accompanying errors and correct D_{1st} choices. The black traces in Figure 3A should therefore lie between the red and blue traces, but this was not the case. In fact, for all subjects, RTs were longest on these single decisions ($p < 0.001$ for all subjects). The force of this observation rests on the assumption that the processes underlying D_2* and D_{2nd} decisions are similar, as they appear to be. Separate fits of the drift-diffusion model to D_2* and D_{2nd} trials show no significant difference in the signal-to-noise and non-decision time parameters ($p > 0.3$ for all subjects and parameters). In fact, the RTs on D_2* resemble the RTs on D_{2nd} when the latter were preceded by the strongest motion on D_{1st} (Figure 3B bottom; $p > 0.41$ all subjects). Intuitively, this is because a D_2* decision, in which the subject only needed to get this decision correct for a reward, is similar to a D_{2nd} decision where the subject would be certain that the first decision was correct (e.g., highest coherence).

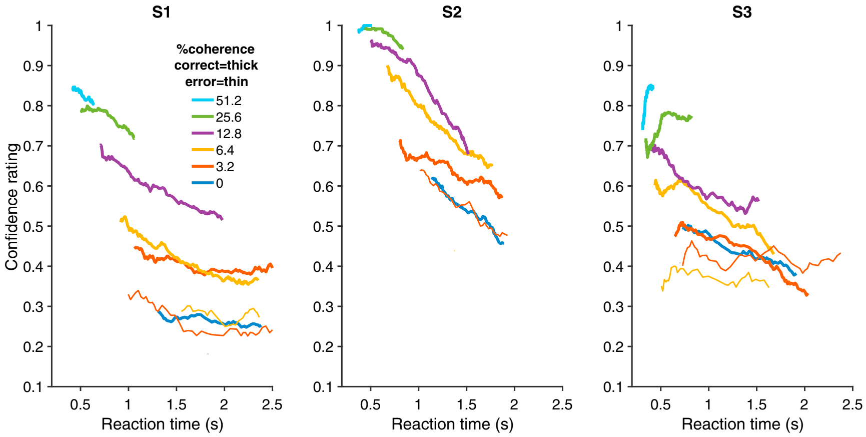

Figure 4. Confidence Ratings for Single First Decisions Vary with Motion Strength and Reaction Time

D_1* and $D_{1st\text{-}catch}$ trials are depicted. Line colors indicate motion coherence; line thickness indicates correct (thick) versus incorrect (thin) trials. All 0% coherence trials are designated as correct for plotting. Data are plotted as a running average (over 70 points for correct trials and 25 points for the fewer error trials).

Third, these sequential effects were only present when the two decisions were part of a single multi-stage decision, rather than just temporally adjacent. We performed the same analysis as in Figure 3B (top) but examined sequential decisions occurring across trials (Figure S3). This showed that the RT on the first decision of one trial is not significantly affected by the coherence of the decision that preceded it, that is, the last decision of the previous trial ($p > 0.19$ for all subjects). This analysis provides reassurance that the effect we report depends on the grouping of the two decisions as part of the same two-stage decision process leading to a reward only if both decisions are correct. From theses analyses (Figures 3B and S3), we conclude that the observed changes in the second of two decisions are not explained by factors common to both decisions or by sequential effects unrelated to performing the multi-stage decision task. Instead, it is an aspect of the experience of the first decision that affects the way the subjects approach the second. We next evaluate our hypothesis that the critical aspect of the first decision is the prediction that the first decision was correct.

Figure 4 shows the confidence ratings obtained after "single first decisions" (see Figure 1). The confidence rating is an arbitrary scaling, but it is significantly influenced by motion strength ($p < 0.0001$, all subjects), RT ($p < 0.0001$), and accuracy ($p < 0.005$), as previously shown [13, 20]. We did not ask the subjects to report their confidence on D_{1st} before making D_{2nd} because we did not want to interfere with the sequential decision. Instead, we estimated their confidence on D_{1st} trials by interpolation, using the coherence, RT, accuracy, and confidence data from the single first decisions (D_1* and $D_{1st\text{-}catch}$; see Experimental Procedures). We have three reasons to believe that this method provided accurate confidence estimates for D_{1st} decisions. First, subjects performed similarly on D_1* and D_{1st} trials. Second, the confidence ratings obtained on D_1* and $D_{1st\text{-}catch}$ trials (Figure 3B) were either indistinguishable ($p > 0.86$ for S1 and S2) or minimally different ($p = 0.044$ for S3; average difference in rating of 0.03; ~5% of the range; see Figure 4). Finally, using leave-one-out cross-validation, our interpolation method accounts for 0.68, 0.56, and 0.46 of the variance in each subject's confidence ratings on D_1* and $D_{1st\text{-}catch}$ trials.

We then asked how the confidence affects the parameters of a diffusion model fit to the choice-RT data from the second decision. We compared six models summarized in Table S2. The best-fitting model (model 3) allows the termination bound, B, to scale with confidence, after controlling for variation in this parameter across days of data collection. This model was superior to alternatives that allowed drift rate and bound to vary by different combinations of session and/or confidence. Model comparison showed that allowing the bound height on D_{2nd} to vary linearly with confidence on D_{1st} had overall very strong support (decrease in Bayesian information criterion [BIC] of 140.9, 46.9, and 4.8 relative to the next best model; designations very strong for S1 and S2 and positive for S3 [26, 27]).

Coherence is a strong determinant of confidence, although the latter is also influenced by choice accuracy and time (Figure 4). We performed a model comparison with the same six models replacing confidence by coherence. The results again favor a bound change (model 3, decrease in BIC of 95.6, 41.3, and 6.0 relative to the next best model). Moreover, confidence was preferred over coherence for this model for two of the three subjects (ΔBIC in favor of confidence of 54.1, 8.7, −4.9). The weak support for coherence (S3) is probably explained by our own limited ability to predict confidence for this subject (see the cross-validation exercise above). Combining BICs across subjects lends strong support for confidence (ΔBIC 58.0; see Experimental Procedures).

Figure 5 depicts the model's change in bound height as a function of the estimated confidence. The dashed line shows the best-fit solution, which is based on the individual trials. To examine our model assumption that bound height varied linearly with confidence, we also fit a model in which we grouped the trials into seven quantiles based on the confidence estimates of D_{1st} (approximately 495 trials per quantile) and allowed the bound height to vary for each quantile when fitting the drift-diffusion model to D_{2nd}. The error bars are standard errors of the estimate of the percentage change in B relative to the average bound across all trials for the lowest-confidence quantile. The dashed line is not a fit to these points—the points and their standard errors simply support our choice of a linear effect of estimated confidence on the change in B. The fits to accuracy and mean RT for four of the quantiles (odd ones) are displayed in Figure 6. They are reasonably good (R^2 range is 0.87 to 0.99 for RT across subjects and all quantiles). From these analyses, we conclude that subjects adjust their criterion on a second decision by slowing down if they are confident on the first decision, and they do so by adjusting the criterion for terminating a decision. This conclusion is further supported by an analysis of the 0% coherence D_{2nd} and D_2* trials, in which only the bound height

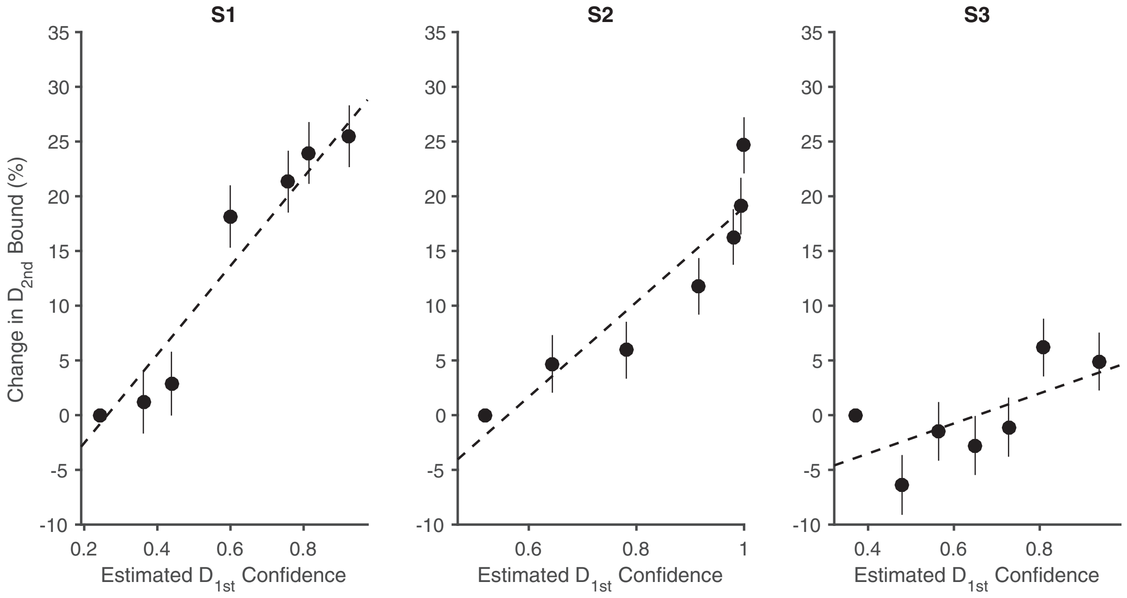

Figure 5. Estimates of the Change in the Bound on the Second Decision as a Function of the Estimated Confidence about the First Decision
The dashed lines depict the best model (model 3). The circles are obtained from a model fit separately to seven quantiles (collapsing over sessions), based on the estimated confidence from the D_{1st} decision. The changes in bound are relative to the lowest-quantile data point (hence no error bar). The dashed line is displaced to match the mean of the quantile fits, as its offset but not slope is arbitrary. See also Figure S4 and Table S2. Error bars indicate SE.

(and non-decision time) determines reaction time. We estimated the bound heights for these second decisions for four quartiles based on D_{1st} confidence and found they were very similar to those from our best-fitting model (Figure S4).

The finding that confidence on one decision affects the bound on the next might explain why subjects adopted conservative bounds on their first decisions (i.e., slow speed, high accuracy; compare Figure 2 and Figure 3) compared with their second. This seems like a sensible strategy because first decisions begin with no sense of futility. They are like second decisions made with the highest confidence on D_{1st}, or like the D_2* control. To examine this further empirically, we examined the mean RTs on 0% coherence motion trials; on these trials, decision time is primarily determined by bound height [28]. As expected, for all subjects, the mean RT from D_{1st} decisions was similar to the mean RT on D_2* decisions (Figure S5; $p > 0.11$ for each subject), which naturally start with highest confidence.

The previous results indicate that subjects adjusted the criteria for terminating a second decision based on confidence in the first decision. The scheme in Figure 7A conveys an intuition for why this may be a sensible strategy. Allocating more time to D_{2nd} (i.e., by changing the termination criteria) increases the reward expected from solving the task correctly (dashed lines). When the confidence in the first decision is low (green), the expected reward plateaus at a lower value than when confidence is high (black), because the expected reward is contingent on having responded correctly to the first decision. The optimal policy (solid line) must balance the marginal benefit expected by deferring the decision against the cost of time and effort (dotted line). Because the marginal benefits are lower following low-confidence first decisions, the optimal decision time for D_{2nd} is shorter if the confidence in D_{1st} is low. This explanation is simple and intuitive, but it is only an approximation of the optimal policy, assuming a cost of time in our task.

We used dynamic programming (see Supplemental Experimental Procedures) to determine whether and how a rational decision maker would adjust the bounds for the second decision of a double-decision task if there is a cost associated with the passage of time. There are many ways in which time can be penalized in such tasks. For simplicity, we chose to examine the normative solution that maximizes reward rate. As has been shown previously for tasks with a single decision, maximizing reward rate [4] or an arbitrary utility function [29] requires adjusting the height of the bound with elapsed time. These previous studies have only considered single decisions, so we extend their framework to our multi-stage decision in which we examine how the bounds should be set given the confidence in D_{1st}.

The normative model prescribes that higher confidence in the D_{1st} decision should lead to higher bounds for the D_{2nd} decision (Figure 7B). The most notable effect of the confidence in D_{1st} is a change in the offset of the bounds for D_{2nd}, without a strong influence on the shape of the bounds. The psychometric and chronometric functions shown in Figure 7C were derived from simulations of diffusion processes using the dynamic programming solution (Figure 7B). Note the resemblance to the corresponding curves in Figure 3A. Specifically, accuracy and RTs were lowest following incorrect first decisions, intermediate following correct first decisions, and highest when the first decision was bypassed. The range of these effects is comparable to what was observed in the behavioral data. To examine the generality of this solution, we also derived the optimal bounds for a range of time costs (equivalent to changing the slope of the cost-of-time lines in Figure 7A) and found qualitatively similar patterns

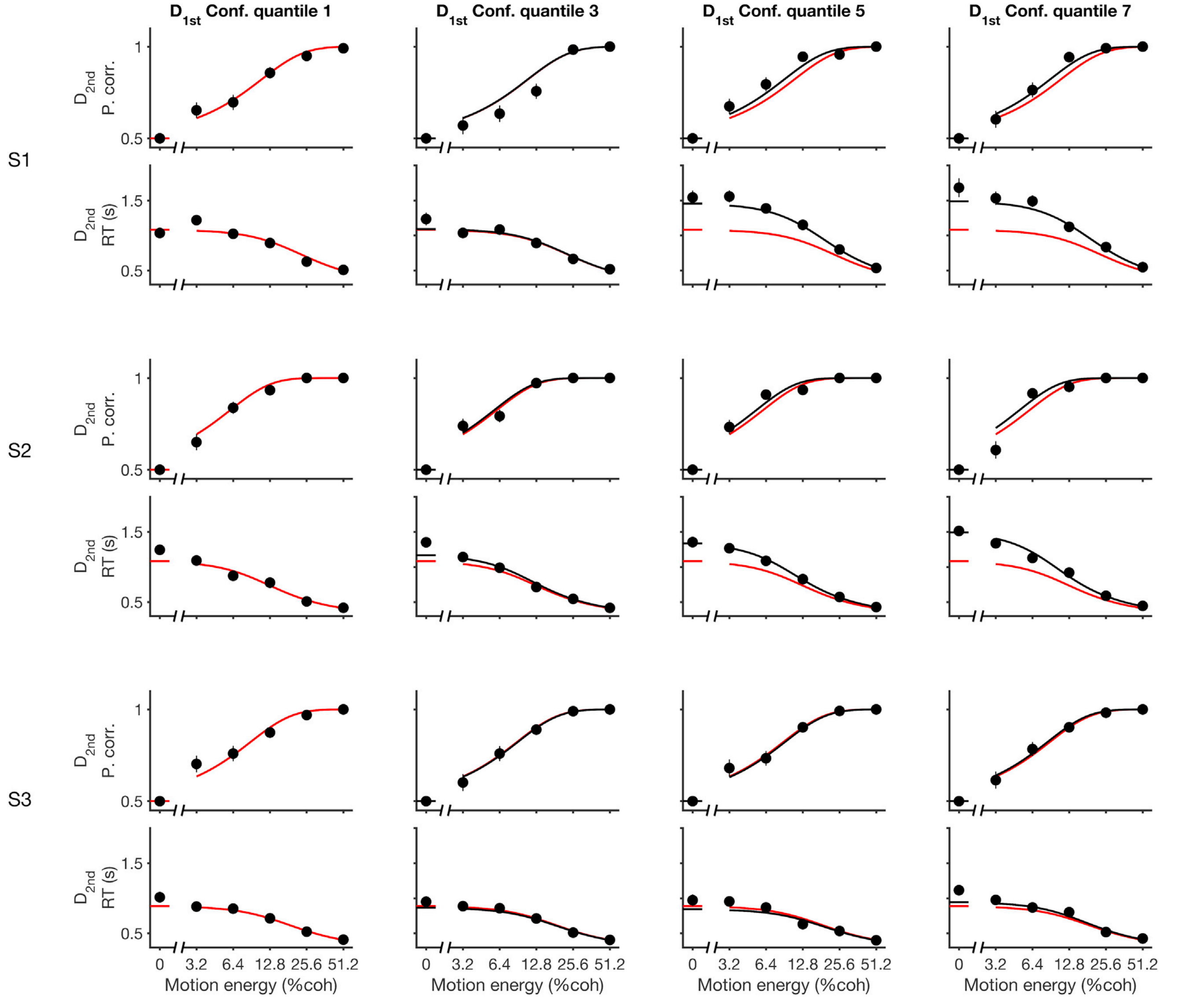

Figure 6. Variation in the Bound Based on D_{1st} Confidence Explains the Accuracy and Reaction Time of D_{2nd} Choices

As in Figure 5, data were split into seven quantiles based on the estimated confidence in the first decision. The columns show the 1st, 3rd, 5th, and 7th quantiles (lowest to highest). Fits (solid lines) incorporate the change in bound for corresponding quantiles from Figure 5. The red lines are the fits to the lowest quantiles of confidence repeated on the other plots for comparison. Error bars indicate SEM. See also Table S2.

of bound changes (data not shown). Therefore, all that is needed for the pattern of bound changes we observe to be rational is a cost of time and a benefit of points. The agreement is only intended as qualitative because the analysis in Figure 7 ignores many complexities in the actual task. Our results do indicate, however, that the strategy exercised by our three subjects—adjusting the bound height for D_{2nd} based on the confidence about D_{1st}—is indeed rational.

DISCUSSION

We have shown that human decision makers are capable of adjusting their speed-accuracy trade-off on the fly based on the recent experience of a decision in a multi-stage decision task. The task is representative of a class of multi-stage decisions in which the outcome depends on all steps along the way. More elaborate cases arise in problem solving (e.g., reasoning step by step) and navigating an uncertain environment. The task we studied is obviously a simple example and is thus capable of bearing on only a fraction of what these more complex endeavors entail. Its main advantages are the consilience with neurobiology and conformance with sequential sampling models, based on biased random walks [1, 30] and drift diffusion [2, 15, 16].

The RTs and choice accuracy in the present dataset were well described by a parsimonious version of bounded evidence accumulation, which we leveraged to gain insight into the mechanism through which subjects used the experience from the first

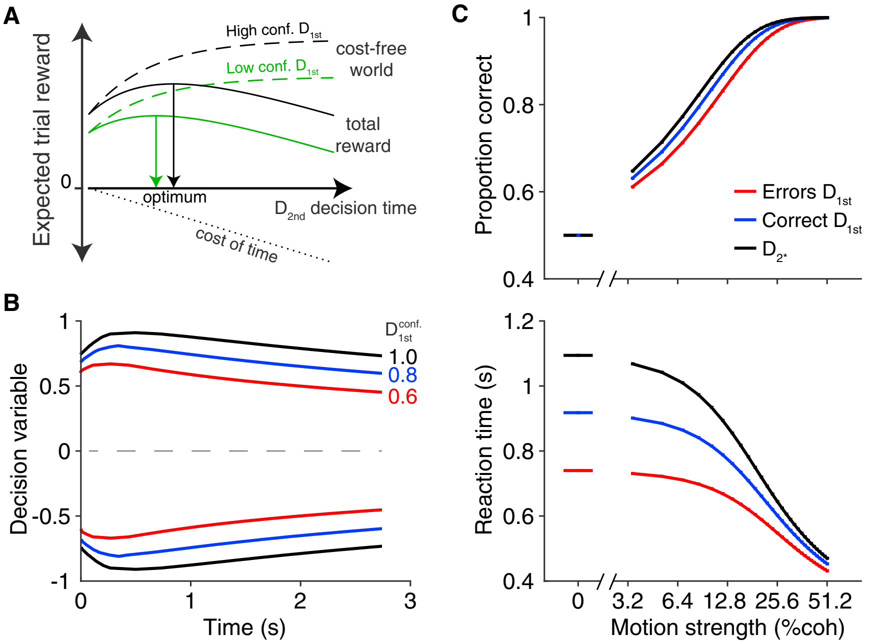

Figure 7. Normative Model for D_{2nd}

(A) Schematic illustration of the impetus to change the strategy on a second decision, based on the confidence in the first. The expected reward after the D_{1st} decision increases monotonically with viewing duration (dashed lines). The expected reward is lower when D_{1st} confidence is low (compare black and green dashed lines). With a cost on time (dotted black line), the total reward (solid lines) has a maximum corresponding to the optimal decision time, which is longer following high-confidence D_{1st} decisions. Although this schematic provides an intuition for our results, we used dynamic programming to derive the optimal solution to maximize reward rate.

(B) Optimal time-dependent bounds from dynamic programming show that the bound height for D_{2nd} increases with D_{1st} confidence.

(C) Model simulation of accuracy and reaction time for second decisions using the optimal bounds in (B). For comparison to Figure 3, the three levels are comparable to a D_2* decision (black: full confidence of 1.0), correct D_{1st} (blue: high confidence of 0.8), and an error on D_{1st} (red: low confidence of 0.6).

decision to alter their strategy on the second. We found clear evidence that subjects adjusted their stopping criterion to allow for more evidence acquisition when they began the second decision with high confidence that their first decision was correct (and hence a greater chance of being rewarded for answering the second decision correctly). The model comparison clearly favored this mechanism over its main alternative, which would posit a boost in signal to noise via concentration of attention and/or noise decorrelation [31–33]. We cannot rule out the possibility that a change in attention occurs, but the pattern of changes in RT and accuracy are explained by a change in the bound height, and the quality of the fits leaves little room for further improvement.

Confidence in the first decision is associated with other factors controlled by the experimenter (e.g., motion strength) or associated with the decision process (e.g., RT and accuracy). These factors are therefore associated with the change in decision bound on the second decision. We reason, however, that they are mediated by the prediction that the first decision was successful, as this establishes the upper bound for joint success in the multi-stage decision. The model comparison lends empirical support for this interpretation, assigning our derived confidence estimates better leverage than motion strength, but that is almost beside the point. Had we found that coherence and our confidence estimates were equivalent, the effect of coherence would be mediated by a process that effectively predicts the probability that D_{1st} is correct—that is, infers confidence. The neural mechanism through which confidence affects the bounds of a subsequent decision is not presently known, but the result highlights the importance of a process that would allow an inferential operation to control another process (e.g., termination criterion).

The particular strategy adopted by our subjects was rational in the sense that it balances success rate against time costs. As we show in Figure 7A, it is wise to integrate for longer on a second decision if one enters that decision with higher confidence that the first decision was correct. This is because the expected final reward, given a correct second choice, increases with the confidence of the first decision, making the extra time invested worthwhile. Conversely, if one has low confidence or even believes that an error was made on the first decision, there is little point spending time on the second decision. We support this point with the modeling exercise in Figures 7B and 7C, but we do not claim that our subjects behaved optimally or that the exercise captures optimality itself. It seems likely that the time costs include opportunity costs or effort of attending the stimulus as well as overall success rate. Nevertheless, we show that increasing the bound with confidence can arise from a normative model (Figures 7B and 7C), suggesting that it is at least a rational strategy.

We focused on the change in the second of two decisions because we were interested in the possibility that confidence would furnish the evidence, as it were, to adjust the controlling parameters of the second decision. We observed little change in the strategy that our subjects applied on the first decision, whether they thought it was the first of a sequence or the only decision they would make (Figure 2). We do not believe that this will hold in general. Clearly, decision makers adopt a different trade-off between speed and accuracy in different contexts. What we have established is that they can do this in a flexible manner that changes over the time course of one second or less.

In the brain, bounds have their signature in a stereotyped level of neural activity at a short latency before the reaction time [15, 24]. This stereotyped level does not depend on RT or the speed-accuracy regime [34, 35]. The brain instantiates a change in bound height by controlling the starting point of the accumulation and adding a time-dependent signal to the accumulated evidence [35, 36]. This is possible because the decision is rendered via a race between two processes, one that

accumulates evidence, say, for up and against down, and another that accumulates evidence for down and against up [37, 38]. These races can adjust the effective bound height by adding the same time-dependent quantity to both accumulators, termed "urgency" [36]. Upon this background, we speculate that the neural mechanism underlying our main effect links confidence in D_{1st} to a change in urgency. Both the readout of confidence and the construction of the urgency signals seem to necessitate structures beyond those that represent the accumulation of evidence. The striatum is likely to a play a role in either or both of these processes [39–41].

The present findings expand our appreciation for the role of confidence in a decision. Confidence is naturally portrayed as a metacognitive assessment—an evaluation of the decision process itself—leading to a belief or rating or a prediction of reward. As such, confidence can be expressed as a choice, for example to postpone action on a decision [42] and to obtain more data or a small but certain reward [11, 43]. The present finding demonstrates that confidence can act as a bridge, linking the outcome of one decision to the strategy applied on a subsequent decision (see also [6]). The process is in some ways like a decision, only instead of deliberating toward a commitment to a proposition among alternatives, it is toward the adoption of a policy—here the relative value of speed versus accuracy.

EXPERIMENTAL PROCEDURES

Four naive subjects (three female and one male) between the ages of 22 and 25 participated in the study. The Cambridge Psychology Research Ethics Committee approved the experimental protocol, and subjects gave informed consent. One of the subjects was excluded from the analyses based on poor task performance (see below). All subjects had normal or corrected-to-normal visual acuity and had no previous experience with random dot motion displays. Prior to participation, they were informed that there was a fixed payment per session. Subjects completed 10–15 sessions. The duration of test sessions (excluding breaks) was 62.0 ± 1.0, 64.6 ± 6.3, and 58.7 ± 0.9 min (mean ± SE) for subjects S1, S2, and S3, respectively.

Apparatus

Subjects were seated in a dimly lit room in front of a 17" Sony Multiscan G200 FD Trinitron CRT monitor (1024 × 768 resolution, 75 Hz refresh). Psychophysics Toolbox [44] and Eyelink Toolbox for MATLAB [45] were used to display images and record eye movements using an EyeLink 1000 (SR Research) in monocular mode at a sampling rate of 1000 Hz. A headrest and chinrest ensured a viewing distance of 42 cm.

Stimulus

Subjects discriminated the direction of motion of dynamic random-dot motion stimuli [46] presented within a circular aperture with a diameter subtending 4° of visual angle. The dots were displayed for one frame (13.3 ms), and then three frames later a subset of these dots were displaced in the direction of motion while the rest of the dots were displaced randomly. Thus, the positions of the dots in frame four, say, could be correlated only with dots in frames one and/or seven but not with dots in frames two, three, five, and six. When displacement made a dot move off the boundary, it was replaced randomly on the opposite boundary in such a way that the coverage of the aperture had on average uniform density. The dot density was 17.9 dots/deg^2/s, and displacements were consistent with a motion speed of 7.1 deg/s. The difficulty of the task was manipulated through the coherence of the stimulus, defined as the probability that each dot would be displaced as opposed to randomly replaced.

Procedure

The majority of trials were *double-decision* trials in which subjects made two discrimination decisions: a left-right decision (D_{1st}), indicated by a leftward or rightward saccade, followed by an up-down decision (D_{2nd}), at the new fixation location to reach one of four final-choice targets (Figure 1A). Critically, for success both decisions needed to be correct. We used a point system to encourage subjects to get both decisions correct. They received no points if either decision was incorrect. We paid subjects for the session, but not based on points, as in general our subjects are self-motivated to accrue points.

The spatial features of the task are not essential. We chose different directions of motion for the two decisions to avoid a tendency to repeat or alternate directions, and we found in pilot experiments that some subjects found the task more natural when they navigated around the screen with linked decisions. In particular, it made the sequence more apparent as a unit than repeating a stimulus in the same location.

At the start of a double-decision trial, a fixation point (blue circular disc, diameter 0.42°) appeared centrally with two choice targets (identical to the fixation point) left and right of the central point (6° eccentricity). In addition, final-choice targets were present at the four possible target locations (white squares with side length 0.42°) above and below the lateral choice targets. After a random delay, sampled from a truncated exponential distribution (range 0.3–1.0 s; mean 0.57 s), the first motion stimulus appeared at the fixation position. Subjects judged the direction of the motion (left versus right) and made an eye movement to the corresponding lateral choice target when ready. Critically, when the movement was initiated—that is, the eye was more than 2.8° from the central point—the random-dot stimulus was extinguished. After fixation had been established at the lateral choice target (defined as within 2.2° from the target center) and a further delay (same distribution as that of the delay before the first decision, which ensured full integration of the first stimulus [17]), the second motion stimulus was presented at the chosen lateral choice target and the subject made a second decision (up versus down) indicated by an eye movement to a final-choice target above or below the stimulus. Again, on saccade initiation the stimulus was extinguished.

On reaching the final-choice target, the chosen target filled. After 0.5 s delay, a bar appeared (5° above the chosen target) within an empty horizontal rectangle on the screen. Subjects provided a confidence rating by rotating a knob (Griffin PowerMate) with their hand so as to adjust the length of the bar to show how confident they were that the final-choice target was correct. The bar's length varied as they rotated the knob, and a number between 0 to 100 was displayed above the bar that corresponded linearly to its length. Subjects were asked to indicate their confidence that the final chosen target was correct (i.e., out of four possible choice targets) by adjusting the knob and pressing a key. Text was displayed at the two ends of the rectangle with "no clue" (at 0) and "absolutely sure" (at 100). After the confidence rating, subjects received auditory feedback about whether they had chosen the correct target.

On each trial, the stimulus coherences were selected randomly and independently for each decision from the set 0%, ±3.2%, ±6.4%, ±12.8%, ±25.6%, and ±51.2%, where negative coherences correspond to leftward/upward motion and positive coherences to rightward/downward motion. On the 0% coherence trials, the direction that would be rewarded was chosen randomly.

Three additional trial types were used (Figure 1, last three rows) in which only a single motion discrimination decision was made and subjects again gave a confidence rating (that they had chosen the correct choice target out of the two options) before they were told whether their decision was correct. On *single first decision* trials (designated D_1*), two choice targets were placed where the lateral fixation points would have been, indicating that the subject would make only a single left-right decision. On *single second decision* trials (designated D_2*), only the final-choice targets on the left or right of the screen were displayed, and the initial left-right motion discrimination was not required. Subjects simply made a saccade to the left or right fixation point before making the single second decision. Finally, we included *single-decision catch* trials (designated $D_{1st\text{-}catch}$), in which subjects thought they would make two decisions (the display was identical to double-decision trials) but were instead presented with a D_1* trial: a confidence rating was asked after the first decision, and there was no second decision. These trials allowed us to compare confidence on single-decision trials with confidence on the first decision on a double-decision trial.

A block of trials consisted of all combinations of double-decision coherence pairs (11 first-stimulus coherences × 11 second-stimulus coherences) and each coherence for the other three trial types (11 coherences for each, making

33 trials), making 154 trials in total. Subjects completed nine sessions (on separate days) and performed four blocks in each session. Stimuli in the first block of a session were all unique. Stimuli in the second block were mirrored versions (horizontally or vertically as appropriate) of the stimuli in the first block. The third and fourth blocks were identical to the first two blocks. The order in which trials were presented was randomized in all blocks. To motivate subjects after each block, their percentage performance over the last block was displayed.

All subjects received extensive training over a number of days on the motion task, in three phases: (1) D_1* trials with computer-controlled variable-duration viewing and no confidence ratings (864 trials completed in a single session), (2) D_1* and D_2* trials without confidence ratings (864 trials per session until choice and reaction times appeared stable; three sessions for subjects S1 and S2, and five sessions for S3), and (3) double-decision trials (99 trials without confidence ratings followed by 154 with confidence ratings). All training was completed before the nine experimental sessions were run.

We required subjects to have sufficient perceptual skills and motivation to perform the task. One subject became unmotivated as the sessions proceeded (failing to turn up for sessions), and an analysis of his first three sessions showed that he also had very strong response bias (e.g., 90% upward responses at 0% coherence trials), so he did not continue in the experiment and we excluded his data from analysis.

Analysis

For each trial, we recorded the choice and reaction time (RT; time to movement initiation from start of motion stimulus) for each decision as well as the final confidence rating (which we divided by 100 so as to be on a 0–1 scale).

We refer to the two decisions of the double decision as D_{1st} and D_{2nd} to distinguish them from single first and single second decisions (D_1* and D_2*, respectively) and the decision made on a single-decision catch trial $D_{1st\text{-}catch}$.

To examine whether accuracy on D_{2nd} is affected by the coherence on D_{1st}, we performed logistic regression on D_{2nd} choices as a function of coherence on D_{2nd},

$$P_{right}(D_{2nd}) = [1+\exp(-(k_1+k_2\,coh_2+k_3\,coh_2|coh_1|))]^{-1},$$

with the null hypothesis that $k_3 = 0$.

To examine whether accuracy on D_{2nd} is affected by accuracy on D_{1st}, we used a chi-square test with Yates correction. To compare reaction times between conditions, we performed ANOVAs on individual subjects with reaction time (individual trials) as a function of condition and absolute coherence (as a categorical variable).

To examine whether the confidence rating on a first decision depended on whether subjects expected to make second decision, we performed ANOVAs of confidence rating with factors of trial type (D_1* versus $D_{1st\text{-}catch}$), motion strength (six levels), and accuracy of D_{1st} (correct versus error). To examine whether the individual confidence rating on a first decision depended on the trial's coherence, RT, and accuracy, we performed an ANOVA on the confidence ratings with categorical factors of unsigned coherence and accuracy, and linear factor RT.

By design, our task does not introduce an interruption between the first and second decision (D_{1st} and D_{2nd}). Thus, we did not solicit confidence reports for D_{1st} and instead estimated these ratings using the D_1* and $D_{1st\text{-}catch}$ trials. For each D_{1st} decision, we selected a fixed number (k) of D_1* and $D_{1st\text{-}catch}$ trials for the same coherence and accuracy (error versus correct) that were closest to the D_{1st} RT and averaged the corresponding confidence ratings (k-nearest neighbor interpolation). We chose k = 30 for correct trials and k = 15 for error trials because errors were less frequent than correct responses. This allowed us to generate estimates of confidence for all D_{1st} trials to examine how the confidence affected D_{2nd} on the same trial.

Naturally, confidence can vary even for trials with the same motion strength, choice, and reaction time. We therefore examined the predictive power of our approach with leave-one-out cross-validation on D_1* and $D_{1st\text{-}catch}$ trials. For each D_1* and $D_{1st\text{-}catch}$ trial, we used the data with the same coherence and accuracy (error versus correct) to predict the confidence on that trial (leaving that trial out of the dataset for the k-nearest neighbor). We repeated this for all of the trials so that we have a leave-one-out prediction of confidence for each trial as well as the actual confidence rating on that trial. We report the fraction of variance explained from these trials.

Model

We used a variant of the drift-diffusion model [23, 47] to explain the proportion of choices and reaction times. The model posits that evidence accumulates from zero until it reaches an upper or lower bound (±B), which determines the initial choice and decision time. The increments of evidence are idealized as normally distributed random variables with unit variance per second and mean $\kappa(C+C_0)$, where C is signed motion strength (specified as the proportion of dots moving in net motion direction, positive = rightward/downward and negative = leftward/upward motion); κ, B and C_0 are free parameters. The parameters B and κ explain the trade-off between speed and accuracy of the initial choices; C_0 is a coherence offset, which explains bias (if any) for one of the choices (starting point bias versus drift bias; see Supplemental Experimental Procedures). The RT incorporates additional latencies, termed the non-decision time (t_{nd}), from stimulus onset to the beginning of the bounded accumulation process and from the termination of the process to the beginning of the motor response.

To fit the accuracy and reaction time of the D_{2nd} and D_2* choices, we minimized the negative log likelihood, using Bernoulli distributions for the choices and Gaussian distributions for the RTs. For analytic simplicity (see below), we used a flat bound (i.e., stationary rather than collapsing), which does not capture the shape of the RT distributions and the mean RT on error trials [4]. Therefore, for the RT component of the response likelihood for each trial, we used only the model's predicted mean RT and used the associated standard deviation from the data for correct trials for the same coherence. Absent bias, correct choices would be rightward choices for positive coherences, leftward choices for negative coherences, and all choices for 0% coherence. In general, these are the direction of the more numerous choices at each coherence, including 0. In practice, we identified the correct trials, when fitting RT, by finding the point of subjective equality in a simple logistic fit to choice and selecting rightward choice trials when $p_{right} > 0.5$ and leftward choice trials for $p_{right} < 0.5$. We did not use the logistic fit to estimate C_0. We optimized using the MATLAB function *fmincon* using analytic gradients.

We used this parsimonious version of the bounded evidence accumulation model, which employs stationary (i.e., flat) bounds. We recognize that the normative prescription for terminating bounds in our experiment incorporates non-stationary (collapsing) bounds [4]. We did not incorporate this degree of complexity in our main model fits in order to reduce complexity and to focus on a single bound parameter (i.e., bound height). This strategy also allowed derivation of model gradients and Hessians allowing efficient and reliable fitting of our models. This practice provides stable estimates of the key parameters (B, κ, t_{nd}).

We examined the stability of the model parameters over the nine sessions and discovered significant variation in the bound parameter (B) and more subtle variation in the other parameters. Furthermore, the values of B covaried for D_{1st} and D_{2nd} (Figure S1). The likelihoods associated with reaction times were calculated using the sample standard deviation separately for each session, subject, and coherence.

To examine how confidence in D_{1st} affected the parameters of the drift-diffusion process accounting for D_{2nd} and D_2* (for which confidence was set to 1), we compared six models (Table S2). We allowed some parameters (B and κ) to vary for each session, whereas other parameters such as C_0 and t_{nd} were shared across all sessions. Table S2 lists the parameters that vary. Here we provide a more intuitive guide. Across the six models, the bound (B) and the signal-to-noise term (κ) can vary, and they can do so in three ways: fixed, by session, and linearly as a function of D_{1st} confidence. Models 1 and 2 are the simplest: either B or κ varies with session, but neither depends on D_{1st} confidence. Models 3 and 4 parallel models 1 and 2, but with an additional variation of B or κ linearly with D_{1st} confidence. Finally, models 5 and 6 allow variation in one parameter, by session, and in the other parameter linearly with D_{1st} confidence. We used the Bayesian information criterion (BIC) to compare the models by controlling for their differing number of free parameters (Table S2). To compute an overall BIC across subjects, we summed the degrees of freedom, number of trials, and log likelihoods for each model.

We also fit a model in which we allowed the bound on D_{2nd} to vary with the session and also to have an additional offset for each of seven quantiles of D_{1st} confidence. This was used not for model comparison but for display purposes, to confirm that our linearity assumption in model 3 (the preferred model) was

reasonable. We were able to use an analytic Hessian to obtain confidence limits on parameters (displayed in Figure 5 and Table S1).

Model Recovery

To validate our selection of model 3 as the preferred model, we examined whether this classification could have arisen if the data had been generated by each of the other five non-preferred models. For each subject, we generated 100 synthetic datasets for each of the five non-preferred models using each subject's best-fit parameters for that model. We generated a synthetic dataset for each model, as follows. For each trial in the experiment with a second decision, we used the subject's estimated D_{1st} confidence and the motion coherence of the stimulus for the second decision, together with the fitted parameters of the model, to generate a synthetic choice (up/down) and RT. The variability in the 100 synthetic datasets for each subject and model arises from the stochastic nature of the drift-diffusion process. We then fit each of the six models to these synthetic datasets. This validation shows that very few of these synthetic datasets had a BIC that was lower than the preferred model type (model 3): 0.8%, 4.8%, and 1.8% for the three subjects. This suggests that had the data come from one of the other models, it is unlikely that we would have misclassified them as model 3.

SUPPLEMENTAL INFORMATION

Supplemental Information includes five figures, two tables, and Supplemental Experimental Procedures and can be found with this article online at http://dx.doi.org/10.1016/j.cub.2016.10.021.

AUTHOR CONTRIBUTIONS

R.v.d.B., A.Z., R.K., M.N.S., and D.M.W.: conception and design, analysis and interpretation of data, drafting or revising the article; R.v.d.B.: acquisition of data.

ACKNOWLEDGMENTS

We thank the Wellcome Trust, the Human Frontier Science Program, the Royal Society (Noreen Murray Professorship in Neurobiology to D.M.W.), Howard Hughes Medical Institute, National Eye Institute grant EY11378 to M.N.S., a Sloan Research Fellowship to R.K., and Simons Collaboration on the Global Brain grant 323439 to R.K. We thank James Ingram for technical support; NaYoung So, Natalie Steinemann, Danique Jeurissen, and Shushruth for comments on the manuscript; and Mariano Sigman for helpful discussions.

Received: August 3, 2016
Revised: September 18, 2016
Accepted: October 12, 2016
Published: November 17, 2016

REFERENCES

1. Link, S.W. (1975). The relative judgment theory of two choice response time. J. Math. Psychol. *12*, 114–135.
2. Ratcliff, R., and Rouder, J.N. (1998). Modeling response times for two-choice decisions. Psychol. Sci. *9*, 347–356.
3. Thura, D., Beauregard-Racine, J., Fradet, C.W., and Cisek, P. (2012). Decision making by urgency gating: theory and experimental support. J. Neurophysiol. *108*, 2912–2930.
4. Drugowitsch, J., Moreno-Bote, R., Churchland, A.K., Shadlen, M.N., and Pouget, A. (2012). The cost of accumulating evidence in perceptual decision making. J. Neurosci. *32*, 3612–3628.
5. Drugowitsch, J., DeAngelis, G.C., Angelaki, D.E., and Pouget, A. (2015). Tuning the speed-accuracy trade-off to maximize reward rate in multisensory decision-making. eLife *4*, e06678.
6. Purcell, B.A., and Kiani, R. (2016). Neural mechanisms of post-error adjustments of decision policy in parietal cortex. Neuron *89*, 658–671.
7. Laming, D. (1979). Choice reaction performance following an error. Acta Psychol. (Amst.) *43*, 199–224.
8. Heitz, R.P., and Schall, J.D. (2012). Neural mechanisms of speed-accuracy tradeoff. Neuron *76*, 616–628.
9. Kolling, N., Behrens, T.E., Mars, R.B., and Rushworth, M.F. (2012). Neural mechanisms of foraging. Science *336*, 95–98.
10. Averbeck, B.B. (2015). Theory of choice in bandit, information sampling and foraging tasks. PLoS Comp. Biol. *11*, e1004164.
11. Kiani, R., and Shadlen, M.N. (2009). Representation of confidence associated with a decision by neurons in the parietal cortex. Science *324*, 759–764.
12. Kiani, R., Corthell, L., and Shadlen, M.N. (2014). Choice certainty is informed by both evidence and decision time. Neuron *84*, 1329–1342.
13. van den Berg, R., Anandalingam, K., Zylberberg, A., Kiani, R., Shadlen, M.N., and Wolpert, D.M. (2016). A common mechanism underlies changes of mind about decisions and confidence. eLife *5*, e12192.
14. Brunton, B.W., Botvinick, M.M., and Brody, C.D. (2013). Rats and humans can optimally accumulate evidence for decision-making. Science *340*, 95–98.
15. Gold, J.I., and Shadlen, M.N. (2007). The neural basis of decision making. Annu. Rev. Neurosci. *30*, 535–574.
16. Shadlen, M.N., and Kiani, R. (2013). Decision making as a window on cognition. Neuron *80*, 791–806.
17. Resulaj, A., Kiani, R., Wolpert, D.M., and Shadlen, M.N. (2009). Changes of mind in decision-making. Nature *461*, 263–266.
18. Burk, D., Ingram, J.N., Franklin, D.W., Shadlen, M.N., and Wolpert, D.M. (2014). Motor effort alters changes of mind in sensorimotor decision making. PLoS ONE *9*, e92681.
19. Moher, J., and Song, J.H. (2014). Perceptual decision processes flexibly adapt to avoid change-of-mind motor costs. J. Vis. *14*, 1–13.
20. Fetsch, C.R., Kiani, R., Newsome, W.T., and Shadlen, M.N. (2014). Effects of cortical microstimulation on confidence in a perceptual decision. Neuron *83*, 797–804.
21. Yu, S., Pleskac, T.J., and Zeigenfuse, M.D. (2015). Dynamics of postdecisional processing of confidence. J. Exp. Psychol. Gen. *144*, 489–510.
22. Pleskac, T.J., and Busemeyer, J.R. (2010). Two-stage dynamic signal detection: a theory of choice, decision time, and confidence. Psychol. Rev. *117*, 864–901.
23. Palmer, J., Huk, A.C., and Shadlen, M.N. (2005). The effect of stimulus strength on the speed and accuracy of a perceptual decision. J. Vis. *5*, 376–404.
24. Smith, P.L., and Ratcliff, R. (2004). Psychology and neurobiology of simple decisions. Trends Neurosci. *27*, 161–168.
25. Pouget, A., Drugowitsch, J., and Kepecs, A. (2016). Confidence and certainty: distinct probabilistic quantities for different goals. Nat. Neurosci. *19*, 366–374.
26. Kass, R.E., and Raftery, A.E. (1995). Bayes factors. J. Am. Stat. Assoc. *90*, 773–795.
27. Jeffreys, H. (1961). Theory of Probability (Oxford University Press).
28. Shadlen, M.N., Hanks, T.D., Churchland, A.K., Kiani, R., and Yang, T. (2006). Bayesian Brain: Probabilistic Approaches to Neural Coding, K. Doya, S. Ishii, A. Pouget, and R.P.N. Rao, eds. (MIT Press), pp. 209–237.
29. Huang, Y., Hanks, T., Shadlen, M., Friesen, A.L., and Rao, R.P. (2012). How prior probability influences decision making: a unifying probabilistic model. In Advances in Neural Information Processing Systems 25 (NIPS 2012), P. Bartlett, F.C.N. Pereira, C.J.C. Burges, L. Bottou, and K.Q. Weinberger, eds. (Neural Information Processing Systems Foundation), pp. 1268–1276.
30. Laming, D. (1968). Information Theory of Choice Reaction Time (Academic Press).
31. Cohen, M.R., and Newsome, W.T. (2008). Context-dependent changes in functional circuitry in visual area MT. Neuron *60*, 162–173.

32. Mitchell, J.F., Sundberg, K.A., and Reynolds, J.H. (2009). Spatial attention decorrelates intrinsic activity fluctuations in macaque area V4. Neuron *63*, 879–888.

33. Cohen, M.R., and Maunsell, J.H. (2009). Attention improves performance primarily by reducing interneuronal correlations. Nat. Neurosci. *12*, 1594–1600.

34. Roitman, J.D., and Shadlen, M.N. (2002). Response of neurons in the lateral intraparietal area during a combined visual discrimination reaction time task. J. Neurosci. *22*, 9475–9489.

35. Hanks, T., Kiani, R., and Shadlen, M.N. (2014). A neural mechanism of speed-accuracy tradeoff in macaque area LIP. eLife *3*, 02260.

36. Churchland, A.K., Kiani, R., and Shadlen, M.N. (2008). Decision-making with multiple alternatives. Nat. Neurosci. *11*, 693–702.

37. Mazurek, M.E., Roitman, J.D., Ditterich, J., and Shadlen, M.N. (2003). A role for neural integrators in perceptual decision making. Cereb. Cortex *13*, 1257–1269.

38. Usher, M., and McClelland, J.L. (2001). The time course of perceptual choice: the leaky, competing accumulator model. Psychol. Rev. *108*, 550–592.

39. Lo, C.C., and Wang, X.J. (2006). Cortico-basal ganglia circuit mechanism for a decision threshold in reaction time tasks. Nat. Neurosci. *9*, 956–963.

40. Ding, L., and Gold, J.I. (2012). Separate, causal roles of the caudate in saccadic choice and execution in a perceptual decision task. Neuron *75*, 865–874.

41. Ding, L., and Gold, J.I. (2013). The basal ganglia's contributions to perceptual decision making. Neuron *79*, 640–649.

42. Kepecs, A., Uchida, N., Zariwala, H.A., and Mainen, Z.F. (2008). Neural correlates, computation and behavioural impact of decision confidence. Nature *455*, 227–231.

43. Hampton, R.R. (2001). Rhesus monkeys know when they remember. Proc. Natl. Acad. Sci. USA *98*, 5359–5362.

44. Brainard, D.H. (1997). The Psychophysics Toolbox. Spat. Vis. *10*, 433–436.

45. Cornelissen, F.W., Peters, E.M., and Palmer, J. (2002). The Eyelink Toolbox: eye tracking with MATLAB and the Psychophysics Toolbox. Behav. Res. Methods Instrum. Comput. *34*, 613–617.

46. Shadlen, M.N., and Newsome, W.T. (2001). Neural basis of a perceptual decision in the parietal cortex (area LIP) of the rhesus monkey. J. Neurophysiol. *86*, 1916–1936.

47. Ratcliff, R. (1978). A theory of memory retrieval. Psychol. Rev. *85*, 59–108.

Current Biology, Volume *26*

Supplemental Information

Confidence Is the Bridge

between Multi-stage Decisions

Ronald van den Berg, Ariel Zylberberg, Roozbeh Kiani, Michael N. Shadlen, and Daniel M. Wolpert

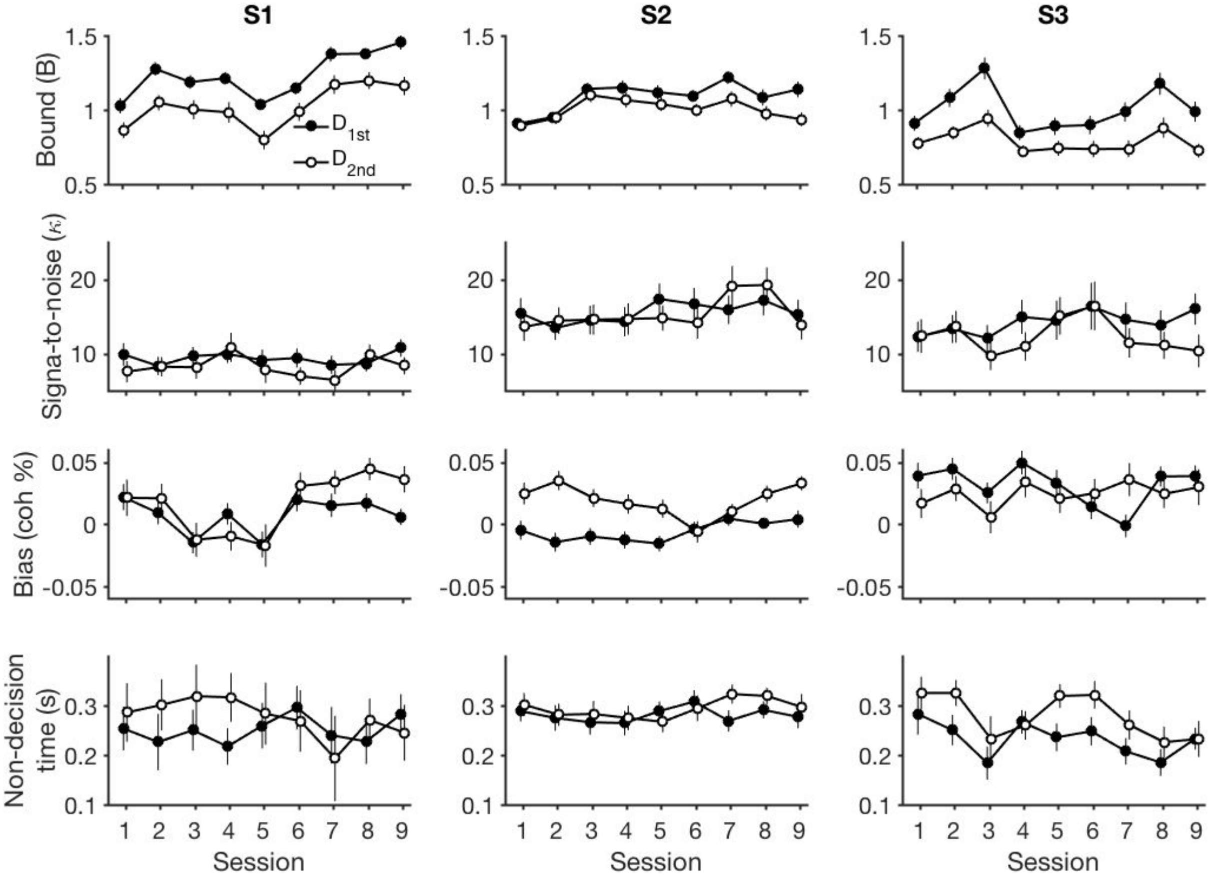

Figure S1. Drift diffusion parameter variation over sessions; Related to Figure 2. The drift-diffusion model was fitted separately to the D_{1st} and D_{2nd} decisions of double-decision trials for each session. Columns are participants and error bars show 95% confidence intervals for parameters estimates.

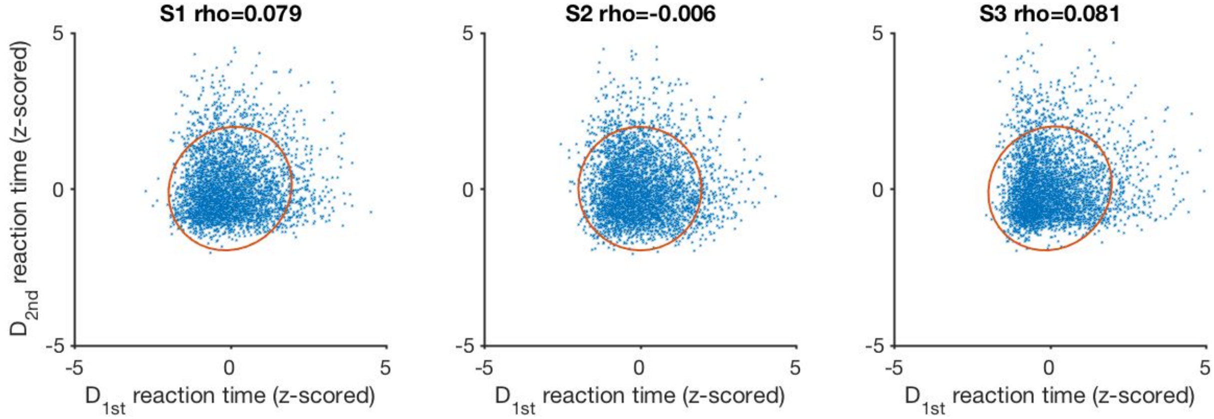

Figure S2. Correlation between reactions times on D_{1st} and D_{2nd}; Related to Figure 3. Reaction times were z-scored within each coherence and session. Also shown are 2-sd principal component ellipses. The correlations are significant for S1 ($p<0.001$) and S3 ($p<0.001$) but not for S2 ($p=0.68$).

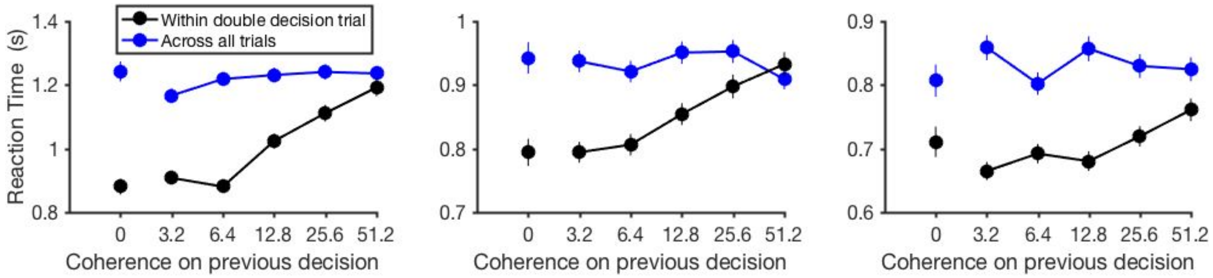

Figure S3 Within vs. across trial effects of coherence on previous decision vs. reaction time on current decision; Related to Figure 3. The black data reproduces Figure 3B (top), showing RT on D_{2nd} and a function of coherence on D_{1st}. The blue data show across-trial effects, showing reaction time on first decision of a trial against the coherence of the last decision on the previous trial. Coherence of the preceding trial decision does not affect the RT of the current decision ($p>0.19$ for all three subjects). Error bars show s.e.m.

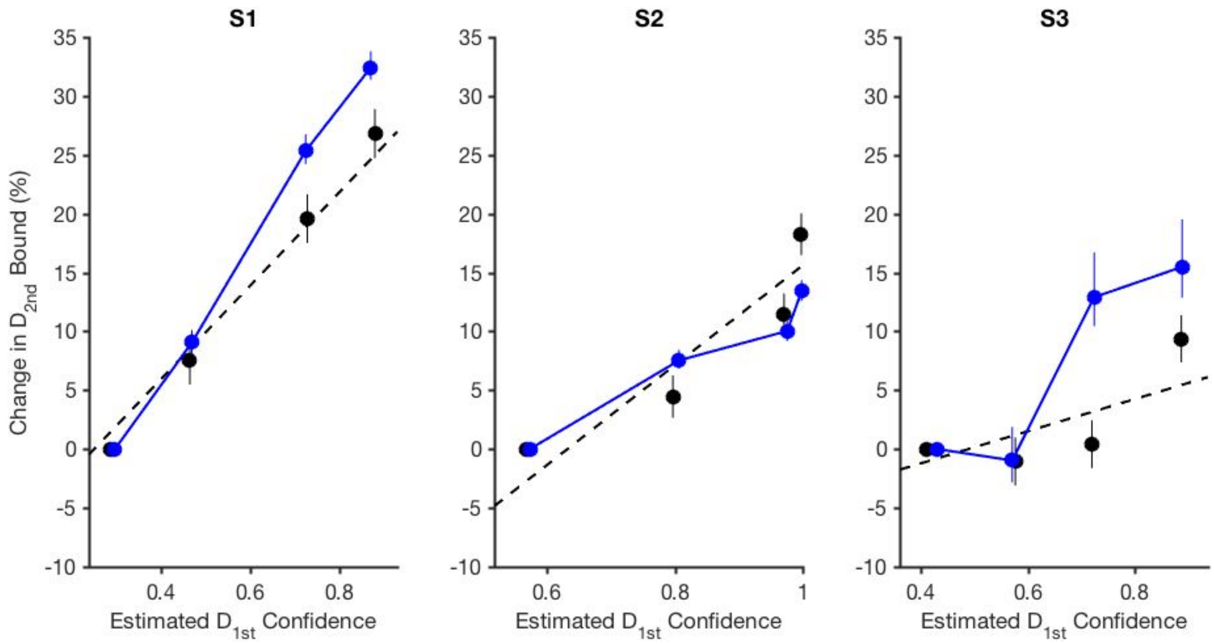

Figure S4. Estimates of the change in the bound on the second decision as a function of the estimated confidence about the first decision; related to Figure 5. The black data and lines are as for Figure 5 but with the model 3 refit for four quantiles. The blue points are derived from the D_{2nd} and D_{2*} 0% coherence trials. On these trials RT is determined by bound height and non decision time ($E[RT]=B^2 + t_{nd}$). For each session we calculated mean RT for each quartile of estimated D_{1st} confidence (quartiles splits across all trials) for these trials and derived the bound height (using t_{nd} =0.280 s, approximately the mean across subjects; Table S2). We plot the across-session mean of the estimated change in second decision bound. Error bars (s.e.) were derived by bootstrapping (1,000 samples).

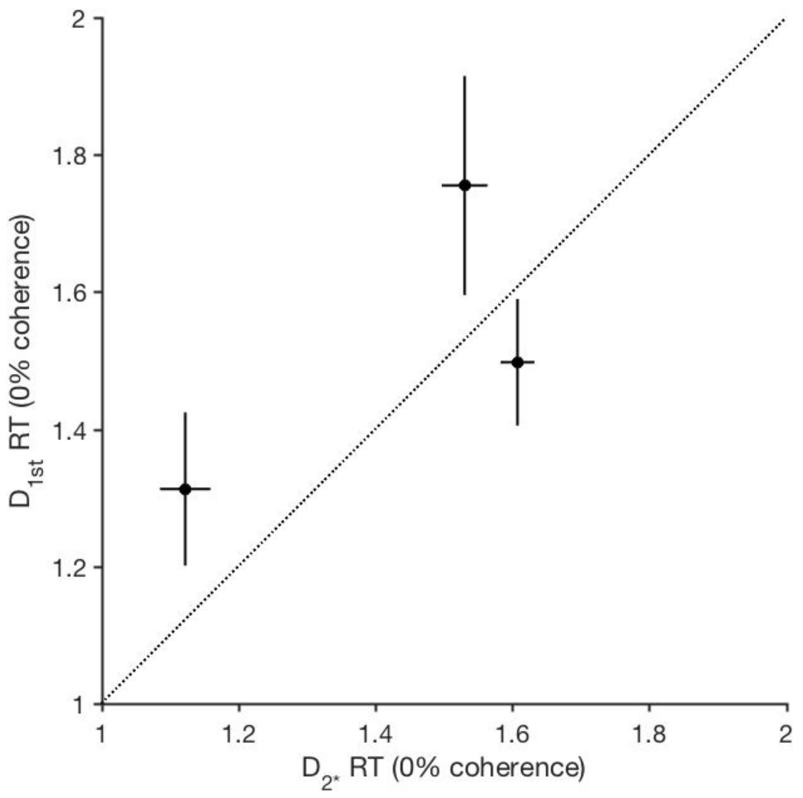

Figure S5. RT on first decisions against second decision; related to Figure 3. Data shows mean (±s.e.) for 0% coherence trials for each subject. The dotted line shows RT equality.

Subject	Trials	Bound (B)	Signal-To-Noise (κ)	Non-decision time (t_{nd}, s)
S1	D_{1*}	1.25 ± 0.03	9.2 ± 0.7	0.242 ± 0.026
	D_{1st}	1.25 ± 0.01	9.1 ± 0.2	0.243 ± 0.008
S2	D_{1*}	1.14 ± 0.02	11.9 ± 0.9	0.250 ± 0.018
	D_{1st}	1.07 ± 0.01	15.1 ± 0.3	0.281 ± 0.004
S3	D_{1*}	1.02 ± 0.04	11.7 ± 0.9	0.193 ± 0.016
	D_{1st}	0.95 ± 0.01	12.4 ± 0.3	0.222 ± 0.005

Table S1. Fitted parameters of the drift-diffusion model; related to Figure 2. The model was fit to single first decisions (D_{1*}) and to the first of two decisions (D_{1st} including $D_{1st\text{-}catch}$ trials). Parameter means are shown ±s.e. For simplicity bias (C_0) was set to zero.

			DOF	ΔBIC from best model		
Model	Bound (B)	Signal-to-noise (κ)		S1	S2	S3
1	$B_{session}$	κ	12	190.83	125.2	5.4
2	B	$\kappa_{session}$	12	409.7	164.5	11.9
3	$B_{session} + \alpha \cdot conf$	κ	13	0.0	0.0	0.0
4	B	$\kappa_{session} + \alpha \cdot conf$	13	364.7	135.9	14.2
5	$B_{session}$	$\kappa + \alpha \cdot conf$	13	140.9	92.1	8.3
6	$B + \alpha \cdot conf$	$\kappa_{session}$	13	227	46.9	4.8
	MLE parameters for Model 3					
		α	1	0.355 ± 0.026	0.379 ± 0.034	0.103 ± 0.028
		$\mathbb{E}[B_{session}]$	9	0.791 ± 0.026	0.678 ± 0.034	0.713 ± 0.025
		κ	1	8.04 ± 0.23	15.44 ± 0.33	11.38 ± 0.33
		C_0	1	0.020 ± 0.002	0.018 ± 0.001	0.026 ± 0.002
		t_{nd} (s)	1	0.283 ± 0.009	0.297 ± 0.003	0.269 ± 0.005

Table S2. Model comparison for fits to the second decision of a double-decision trial (D_{2nd}); related to Figures 5 & 6. The models vary in whether and how they allow the bound (B) and κ for the second decision to vary (see Methods for details). Some models have different levels of B or κ for each of the 9 sessions (subscripts) and others allow these parameter to vary linearly with the predicted confidence from D_{1st} (conf). The degrees of freedom (DOF) of the models and their difference in BIC from the best model are shown. The maximum likelihood estimates of the parameters for the best model (3) are also shown with s.e.

Supplemental Experimental Procedures

Starting point vs. drift bias

We accounted for possible biases by including a bias on drift in the model (the C_0 parameter). However, there is some evidence suggesting that the locus of bias is instead in bound asymmetries (Refs S1, S2), (but see S3) which is equivalent to a starting point bias in our model. We compared these alternatives by fitting all first choices that were part of a double decision (D_{1st}), with either a C_0 (coherence bias) term or y_0 (offset bias) term. For all three subjects, the model with C_0 bias was strongly preferred over the model with y_0 (ΔBIC is 21.8, 21.1, and 183.0 for subjects 1-3, respectively; same as deviance as d.f. are same), which justifies the assumption in our main model. Note that S3 is the most informative subject as bias is small for S1 and S2. We chose to fit with only C_0 to reduce the number of parameters.

Normative model

We used dynamic programming to determine the optimal decision policy for D_{2nd} as a function of the confidence in D_{1st}. By optimal we refer to the decision policy that maximizes reward rate (i.e., maximizing the number of points obtained per unit of time). The goal of this exercise is not to establish that our participants were maximizing reward rate, but to justify their strategy as sensible given a cost of time per trial.

The random dot motion discrimination task can be considered an instance of a class of problems referred to as partially-observable Markov Decision Process (POMDP). The partial observability derives from the fact that (motion) observations provide only ambiguous evidence about the true underlying task state. Following the usual approach, we solve the POMDP casting it as a fully observable Markov decision process (MDP) over the belief states of the agent. We then use dynamic programming to find the policy that maximizes average reward.

Formally, an MDP can be described as a tuple given by (S4):

(i) a non-empty state space S,

(ii) an initial state S_0,

(iii) a goal state S_G,

(iv) a set of actions $A(s)$ applicable in state s,

(v) positive and negative rewards $r(a,s)$ for doing action a in state s,

(vi) transition probabilities $P_a(s'|s)$ indexing the probability of transitioning to state s' after doing action a in state s.

For simplicity, we derive the optimal policy for the second decision assuming that D_{2nd} is informed by the confidence in D_{1st}, without explicitly modeling the decision process for the first decision. Next, we describe how to cast the motion discrimination task as an MDP.

The state s was defined as a tuple $\langle x, t, c_1 \rangle$, where x is the amount of accumulated motion evidence for one direction and against the other (the decision variable). It is positive when the evidence supports one motion direction (say upwards), and negative when it supports the opposite direction. t is the elapsed decision time since the onset of motion for the second decision. c_1 is the probability that the first decision was correct. We assume that c_1 takes a value from the set $C_1 = [0.6, 0.8, 1]$, which corresponds respectively to the average confidence for incorrect, correct and bypassed first decisions. This is a simplification because correct and incorrect decisions are associated with a distribution of confidence values. However, we note that our conclusions do not depend on this simplification as long as the average confidence about D_{1st} is higher for correct than for error trials, which is indeed what was observed in our data (**Figure 2** and **4**). Further, we assume that the probability of eliciting each of the values in C_1 was given by $p_{C_1} = [0.3, 0.5, 0.2]$. Again, our conclusions are robust to changes in these values.

The decision process starts with $x = 0$ (i.e., no accumulated evidence favoring either of the alternatives), $t = 0$ and $c_1 \in C_1$. The distribution over c_1 was implemented with an initial state s_0 that has transition probabilities p_{c_1} to the three states $\langle x = 0, t = 0, c_1 \in C_1 \rangle$.

Three actions were applicable in each state. The decision maker could either terminate the trial by selecting one of the targets (two possible actions), or maintain fixation (the third 'action') to gather additional motion evidence. Defining a deterministic policy entails specifying which action to select in each state.

Transition probabilities $P_a(s'|s)$ indicate the probability of transitioning to s' after performing action a in state s. State transitions are not deterministic because they depend on the momentary motion evidence, which is stochastic even if the motion coherence were known. As in the bounded accumulation model, we assume that the momentary motion evidence follows a normal distribution with a mean that depends linearly on motion coherence, such that over

one second of stimulus viewing the evidence accumulated is, on average, κ.*coh* and the variance of the momentary is equal to 1. For the analyses shown in **Figure 7** we set κ=10.

For a given motion coherence, the probability of transitioning from state $s = \langle x, t, c_1 \rangle$ to state $s' = \langle x', t+\delta t, c_1 \rangle$ is given by:

$$p_{fix}(s'|s, coh) = p_{fix}(\langle x', t+\delta t, c_1\rangle \mid \langle x, t, c_1\rangle, coh) = \mathcal{N}(x'-x|\kappa.coh.dt, \sqrt{\delta t}) \quad (1)$$

where $N(\cdot|\mu, \sigma)$ is the normal p.d.f. with mean μ and standard deviation σ.

Because the decision-maker does not know the motion coherence with certainty, obtaining the transition probability $p_{fix}(s' \mid s)$ requires marginalizing over coherences:

$$p_{fix}(s'|s) = \sum_{coh} p_{fix}(s'|s, coh) p(coh|s) \quad (2)$$

This marginalization requires knowledge of $p(coh|s)$, the probability that the underlying motion coherence is coh given that state s was reached (S5):

$$p(coh|\langle x, t, c_1\rangle) = \frac{1}{\mathcal{Z}} \mathcal{N}(x|\kappa.coh.t, \sqrt{t}) p(coh) \quad (3)$$

where the coherences coh are the discrete set of signed coherences used in the experiment, and Z is the normalization constant which assures that the sum of $p(coh|\langle x, t, c_1\rangle)$ over all motion coherences adds to one. As in the experiment, $p(coh)$ is distributed uniformly over the discrete set of motion coherences.

We assume that the optimal decision-maker maximizes the reward per unit of time. To find the optimal policy, we used value iteration to solve Bellman's equation. For problems that have a recurrent state—which includes decision-making tasks that are organized as a sequence of trials—the problem of maximizing average reward can be recast as a stochastic shortest path problem (or Goal MDP) through the inclusion of an artificial cost-free and absorbing goal state (S4). The intuition behind this simplification is that if we consider a sequence of generated

trajectories in state space, we can divide it into a series of visits to the recurrent state, which is equivalent to the corresponding Goal MDP where the recurrent state is the goal state (S4).

For our task, the Bellman equation takes the form:

$$V(s) = max \begin{cases} Q(s,up) & = p_{c|up}(s)R_c + p_{nc|up}(s)R_{nc} - (t_{other} + t_{nd})\rho \\ Q(s,down) & = p_{c|down}(s)R_c + p_{nc|down}(s)R_{nc} - (t_{other} + t_{nd})\rho \\ Q(s,fix) & = E[V(s')|s] - \rho\delta t \end{cases} \quad (4)$$

where s , s' are states and t_{nd} is the average non-decision time. The time t_{other} is the average time gap between a response to D_{2nd} and the onset of motion for the following D_{2nd}; it includes the time spent on fixations, reporting confidence, responding to the first decision, receiving feedback, etc. For the analyses of **Figure 7**, we chose $t_{nd} = 0.3\ s$ and $t_{other} = 5\ s$. R_c is the reward obtained after a correct response, and R_{nc} is the reward obtained after an incorrect response. As in the experiment, $R_c = 1$ and $R_{nc} = 0$. Reward rate ρ is the reward obtained per unit of time.

$p_{c|a}(s)$ is the probability of being correct after doing action a in state s . For the double decision trials, being correct means solving both decisions correctly. Therefore, $p_{c|a}(s)$ is the probability that the first decision was correct (c_1) , multiplied by the probability that D_{2nd} was solved correctly. The latter can be obtained summing over the coherences for which the action a is the appropriate action. For instance, the action 'up' is the appropriate action for all positive and for half of the 0% coherence trials. Therefore

$$p_{c|up}(s) = p_{c|up}(x,t,c_1) = c_1 \times \left(\sum_{coh>0} p(coh|x,t) + \frac{1}{2} p(coh=0|x,t) \right) \quad (5)$$

Our depiction of Bellman's equation implicitly assumes that choosing a terminal action leads to an absorbing cost-free state. The expectation in $Q(s,fix)$ is an expectation over all future states s' that result from being in s and gathering evidence for an additional time step δt :

$$E[V(s')|s] = \int_{s' \in S} ds' p_{fix}(s'|s)V(s') \quad (6)$$

Because time flows in a single direction, Bellman's equation can be solved by backwards induction in a single pass. However, since we want to maximize the reward rate, which depends

on the policy itself, we perform multiple backwards passes to find the value of ρ through root-finding, bracketing ρ within a sequence of diminishing intervals until the value of the initial state $V(S_0)$ is approximately zero (S4, S6).

Supplemental References

S1. White, C. N., and Poldrack, R. A. (2014) Decomposing bias in different types of simple decisions. J. Exp. Psychol. Learn. Mem. Cogn. 40, 385–398

S2. van Ravenzwaaij, D., Mulder, M. J., Tuerlinckx, F., and Wagenmakers, E.-J. (2012) Do the dynamics of prior information depend on task context? An analysis of optimal performance and an empirical test. Front. Psychol. 3, 132

S3. Moran, R. (2015) Optimal decision making in heterogeneous and biased environments. Psychon. Bull. Rev. 22, 38–53

S4. Bertsekas, D. P. (1995) Dynamic programming and optimal control (Athena Scientific, Belmont, MA)

S5. Moreno-Bote, R. (2010) Decision confidence and uncertainty in diffusion models with partially correlated neuronal integrators. Neural Comput. 22, 1786–1811

S6. Drugowitsch, J., Moreno-Bote, R., Churchland, A. K., Shadlen, M. N., and Pouget, A. (2012) The cost of accumulating evidence in perceptual decision making. J. Neurosci. 32, 3612–3628
